# Targeted gene correction of human hematopoietic stem cells for the treatment of Wiskott - Aldrich Syndrome

**DOI:** 10.1038/s41467-020-17626-2

**Published:** 2020-08-12

**Authors:** Rajeev Rai, Marianna Romito, Elizabeth Rivers, Giandomenico Turchiano, Georges Blattner, Winston Vetharoy, Dariusz Ladon, Geoffroy Andrieux, Fang Zhang, Marta Zinicola, Diego Leon-Rico, Giorgia Santilli, Adrian J. Thrasher, Alessia Cavazza

**Affiliations:** 1 Infection, Immunity and Inflammation Research and Teaching Department, Great Ormond Street Institute of Child Health, University College London, 30 Guilford Street, London, WC1N 1EH UK; 2 SIHMDS-Acquired Genomics, Great Ormond Street Hospital for Children NHS Foundation Trust, Great Ormond Street, London, WC1N 3JH UK; 3 Institute of Medical Bioinformatics and System Medicine, University of Freiburg, 26 Stefan-Meier-Strasse, 79104 Freiburg, Germany

**Keywords:** Genetic engineering, Targeted gene repair, Stem-cell biotechnology, Primary immunodeficiency disorders, Molecular medicine

## Abstract

Wiskott-Aldrich syndrome (WAS) is an X-linked primary immunodeficiency with severe platelet abnormalities and complex immunodeficiency. Although clinical gene therapy approaches using lentiviral vectors have produced encouraging results, full immune and platelet reconstitution is not always achieved. Here we show that a CRISPR/Cas9-based genome editing strategy allows the precise correction of *WAS* mutations in up to 60% of human hematopoietic stem and progenitor cells (HSPCs), without impairing cell viability and differentiation potential. Delivery of the editing reagents to WAS HSPCs led to full rescue of WASp expression and correction of functional defects in myeloid and lymphoid cells. Primary and secondary transplantation of corrected WAS HSPCs into immunodeficient mice showed persistence of edited cells for up to 26 weeks and efficient targeting of long-term repopulating stem cells. Finally, no major genotoxicity was associated with the gene editing process, paving the way for an alternative, yet highly efficient and safe therapy.

## Abstract (web summary)

In recent years, hematopoietic stem cells gene editing has emerged as a promising tool to treat blood disorders. Here the authors develop a CRISPR/Cas9-based genome editing strategy that allows the precise correction of Wiskott-Aldrich Syndrome in vitro and in vivo with high efficiency.

## Introduction

Wiskott–Aldrich Syndrome (WAS) is an X-linked recessive primary immunodeficiency disease characterised by microthrombocytopenia, recurrent infection and complex immunodeficiency. WAS is caused by mutations in the *WAS* gene, which lead to defective WAS protein (WASp) expression or function^1,2^. WASp is a regulator of the actin cytoskeleton and its deficiency disrupts many dependent processes^3^. Without definitive treatment, the prognosis of classical patients diagnosed with WAS remains poor^1,4,5^.

WASp is broadly expressed in hematopoietic cells and, accordingly, full correction of WAS requires the restoration of WASp expression in nearly all hematopoietic lineages. Hematopoietic stem cell transplantation (HSCT) is highly effective but the increased morbidity and mortality associated with HSCT from mismatched donors^6–8^ have prompted the search for alternative therapeutic approaches. Viral vector-based gene addition reduces the risk of alloreactivity while providing a curative option for all patients. Following development of  insertional mutagenesis in WAS patients treated with a γ-retroviral vector^9,10^, subsequent gene therapy clinical trials have utilised a self-inactivating lentiviral vector (LV) with a 1.6-kb fragment of the endogenous *WAS* promoter to regulate WASp expression^11–13^. Patients treated with this LV have shown substantial clinical improvement, with decreased frequency of bleeding and infection episodes and resolution of eczema. However, despite robust correction of T lymphocyte abnormalities, correction of other lineages (platelets in particular) has proved more challenging, reflecting a deficiency in vector construction for reciprocating physiological gene expression. Furthermore, lentiviral vectors carry an intrinsic potential risk of genotoxicity due to their semi-random integration pattern.

Gene editing is an alternative to conventional gene addition therapy and may overcome some of its limitations. Homology Directed Repair (HDR)-mediated integration of a cDNA transgene at specific sequences offers much more control over viral vector site integration and copy number; moreover, targeted knock-in of a cDNA into its endogenous locus improves the likelihood of physiologically regulated gene expression. Recent studies have shown the feasibility of this strategy to tackle primary immunodeficiencies^14–17^. Here, we have developed a CRISPR/Cas9 gene editing platform to knock-in a therapeutic *WAS* cDNA in frame with its endogenous translation start codon in patient-derived hematopoietic stem and progenitor cells (HSPCs), allowing transcriptional regulations from *WAS* regulatory regions. As WAS arises from >300 genetic mutations scattered throughout the *WAS* gene, this strategy ensures correction of all known disease-causing mutations^2^.

## Results

### CRISPR/Cas9-mediated editing of the *WAS* locus in HSPCs

**Fig. 1:**
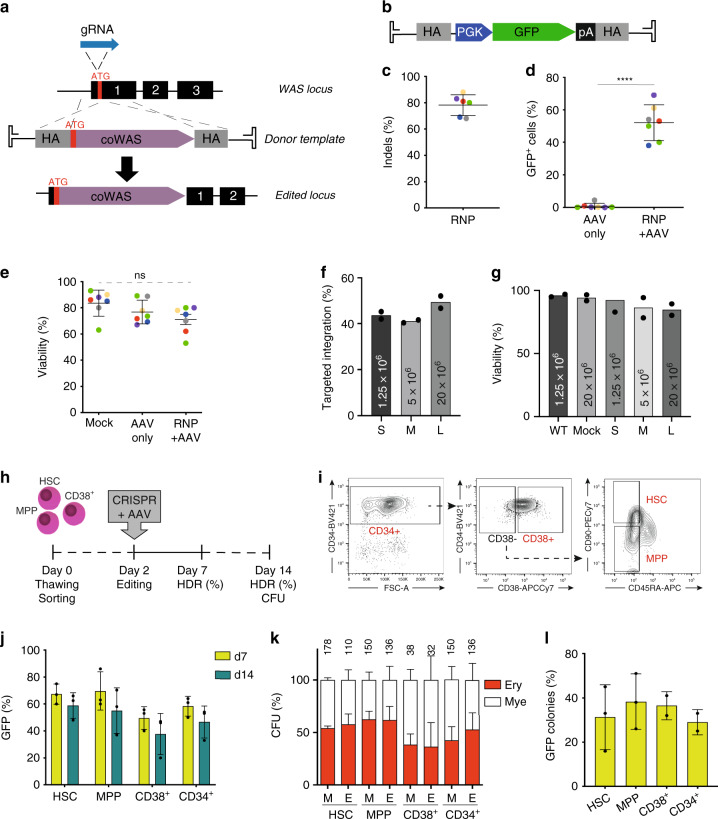
Development of a stem-cell gene editing platform targeting *WAS*. **a** Schematics of the gene editing strategy used to knock-in a codon-optimised (Co) *WAS* cDNA in its genomic locus through HDR (HA homology arms, ATG translational start codon, black boxes exons). **b** For initial protocol set up, an AAV6 donor containing a PGK-GFP cassette was used. **c** HSPCs were electroporated with a Cas9:gRNA complex and INDELs were measured (*n* = 6 experiments, coloured dots represent different PB donors) **d** HSPCs were either electroporated and transduced with the AAV6 donor vector (RNP + AAV) or only transduced (AAV only) and analysed by flow cytometry. HDR can be inferred by the percentage of GFP-positive cells (*n* = 7 experiments; coloured dots represent different PB donors; ****p < 0.0001, two-tailed paired Student’s *t* test). **e** Viability of Mock, AAV-only and RNP + AAV cells was assessed by flow cytometry (*n* = 7 experiments; coloured dots represent different PB donors; NS non significant by ordinary one-way ANOVA with Bonferroni’s multiple comparison test. **f** Rates of HDR and **g** viability obtained with increasing amount of HSPCs (S small, M medium, L large scale) and a clinical-grade electroporator (*n* = 2 experiments from two different PB donors). **h** Sorted HSCs, MPPs, CD34 + CD38+ and unsorted CD34+ HSPCs from three healthy donors were cultured and edited two days after thawing and sorting. Editing rates were assessed at day 7 and day 14 of culture. **i** Gating strategy for the isolation of the different cell populations from CD34+ HSPCs. **j** HDR rates measured by flow cytometry at day 7 and day 14 of culture for each cell population (*n* = 3 experiments from three different PB donors;). **k** Percentage of myeloid (white) and erythroid (red) colonies formed in methylcellulose by Mock (M) and edited (E) cells for each subpopulation. Absolute numbers of clones are shown (*n* = 3 experiments from three different PB donors; NS: *p* > 0.05, as analysed by one-way ANOVA with Bonferroni’s multiple comparison test). **l** Percentage of GFP-positive colonies formed in methylcellulose by edited HSCs, MPPs, CD38+ and CD34+ cells (*n* = 3 experiments from three different PB donors; **p < 0.01, *p < 0.05; paired *t* test). Data are presented as mean ± SD. Source data are provided as a Source Data file.

To mediate the site-specific integration of a *WAS* cDNA in the *WAS* genomic locus (Fig. 1a) we designed different gRNAs targeting the first exon of the *WAS* gene and tested their activity in K562 cells. Allelic disruption (indels formation) rates of up to 45% (32.3 ± 12.5) were achieved with gRNA-1, which was selected for all further experiments (Supplementary Fig. 1A, B). Delivery of the gRNA pre-complexed to Cas9 protein as ribonucleoproteins (RNP) to peripheral blood (PB)-derived CD34+ HSPCs from healthy male donors yielded up to 90% (78.1 ± 7.9) of indels formation, with the highest frequency of allelic disruption being achieved when using a combination of chemically modified gRNA^18^ and high-fidelity (HiFi) version of Cas9^19^ (Fig. 1c, Supplementary Fig. 1C–F). Correction of the genomic break by non homologous end joining (NHEJ) led to either 1 base pair insertion or 4 base pair deletion upstream of *WAS* start codon in the majority of HSPCs, without alteration of the *WAS* coding sequence (Supplementary Fig. 1D, E). To deliver the donor DNA molecule which serves as a template for HDR-mediated repair, we created an AAV6 vector that contains a GFP reporter cassette flanked on each side by sequences with homology to the *WAS* regions surrounding the gRNA’s cut site (Fig. 1c). By RNP electroporation, followed by transduction with the AAV6 donor vector, we observed targeted integration of the PGK-GFP reporter cassette in up to 69% of HSPCs (52.1% ± 10.9), with no significant decrease in cell viability compared to mock-targeted HSPCs (Fig. 1d, e; Supplementary Fig. 1G). Up-scaling of the optimised gene editing protocol for clinical readiness using a clinical-grade electroporator yielded rates of targeted integration similar or higher to those observed at small scale, while preserving cell viability (Fig. 1f, g).

To achieve a life-long benefit from any gene therapy approach, targeting the most primitive population of HSCs with long-term repopulating potential is of paramount importance. To estimate rates of gene editing in different subpopulations of stem and progenitor cells, HSCs, multipotent progenitors (MPPs) and CD38+ committed progenitors were sorted from PB-derived CD34+ HSPCs immediately after cell thawing, using a well-established panel of markers^20^ (Fig. 1h, i; Supplementary Fig. 1 H). Two days post sorting, the three populations, as well as unsorted CD34^+^ cells, were edited by delivery of the Cas9/gRNA RNP and the AAV-PGK-GFP donor vector and rates of targeted integration were determined 5 and 12 days after editing, for a total of 7 and 14 days of cell culture, respectively. HDR-mediated knock-in of the reporter cassette was detected in all cell populations, with a comparable amount of GFP-expressing cells observed at both time points, indicating stable integration of the reporter cassette and lack of negative selection/cytotoxicity of edited cells (Fig. 1j; Supplementary Table 3). Notably, we achieved extremely high levels of targeted integration in the HSC-sorted population, with an average of 67.3% ± 7.5 and 58.8% ± 9.5 GFP^+^ cells at culture days 7 and 14, respectively. To evaluate the capacity of edited stem and progenitor cells to differentiate into multiple lineages, cells were subjected to in vitro colony forming unit (CFU) assays. Edited cells retained their clonogenic potential and produced similar frequencies of erythroid and myeloid cells without lineage skewing compared to controls while yielding similar number of colonies of mock-treated cells (Fig. 1k). Up to 51% of the colonies formed in solid cultures were GFP-positive in all the populations (HSC: 31.3% ± 14.6; MPP: 38.3 ± 12.5; CD38+:36.5 ± 6.3; and unsorted: 29 ± 5.6; Fig. 1l), confirming high frequency of targeted integration in clonogenic cells.

### Functional correction of WAS HSPCs and their myeloid progeny

**Fig. 2:**
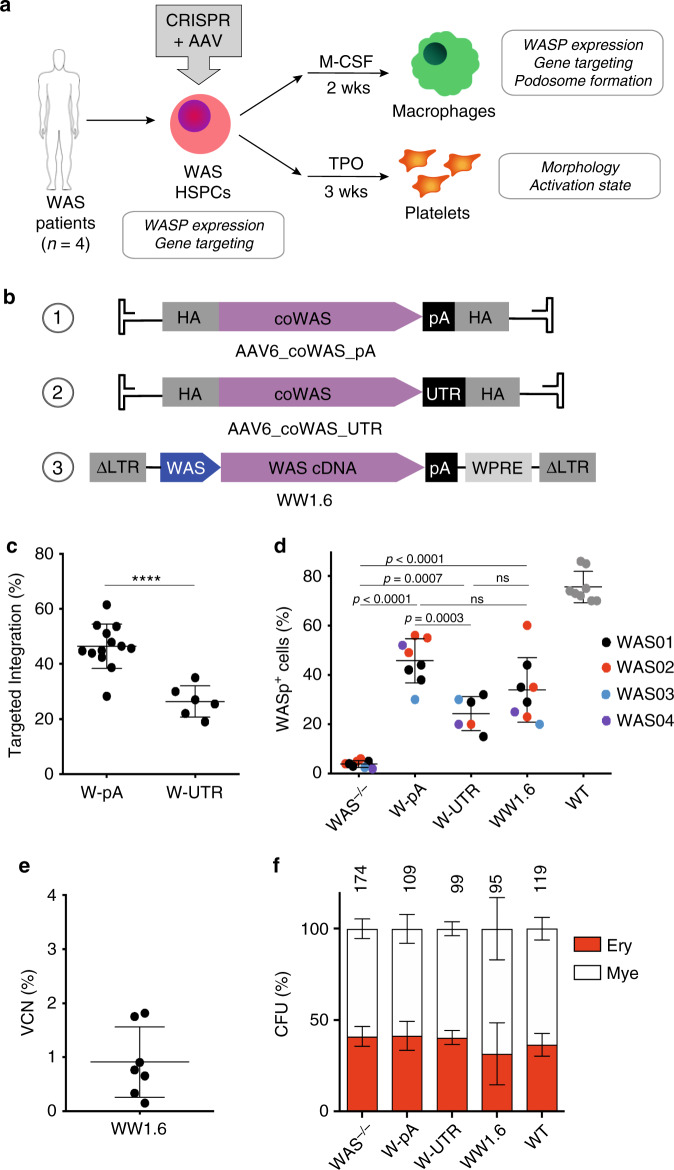
Gene editing of WAS HSPCs. **a** Experimental plan to assess functional correction of the WAS defect in macrophages and platelets by targeting WAS HSPCs. This figure was created using Servier Medical Art templates, which are licensed under a Creative Commons Atrtribution 3.0 Unported License; https://smart.servier.com. **b** Schematic of WAS corrective AAV6 donor vectors containing the coWAS cDNA followed by either a BGH polyA signal (coWAS_pA) or WAS 3′UTR (coWAS_UTR). In parallel, the same cells were transduced with a lentiviral vector (WW1.6) currently used in WAS gene therapy clinical trial (HA homology arms, WAS WAS promoter, LTR long terminal repeats, WPRE woodchuck hepatitis virus posttranscriptional regulatory element). **c** Rates of targeted integration achieved with the two AAV6 donor vectors detected by ddPCR (*n* = 6 and 13 independent experiments for W-UTR and W-pA, respectively, from 4 different donor sources; Asterisks indicate *p*-values: ****p < 0.0001, two-tailed unpaired Student’s *t* test). **d** Quantification of WASp expression in mock, edited, LV-transduced WAS HSPC and healthy HSPCs (WT) by flow cytometry (*n* = 8 independent experiments for all groups except for W-UTR (*n* = 6) from four different donor sources; exact *p*-values are shown in the panel, NS: *p* > 0.05; one-way ANOVA with Bonferroni’s multiple comparison test). **e** Quantification of the number of lentiviral vector copies integrated into the genome of transduced WAS HSPCs (VCN, vector copy number; *n* = 7 experiments from four different donors). **f** Plots representing the percentage of myeloid (white) and erythroid (red) GFP-positive colonies formed in methylcellulose by mock, edited, transduced WAS HSPCs and healthy HSPCs (WT). Absolute numbers of clones derived from each condition are shown (*n* = 4 experiments from four different donors; NS: *p* > 0.05, as analysed by one-way ANOVA with Bonferroni’s multiple comparison test). Data in Fig. 2 are presented as mean ± SD. Source data are provided as a Source Data file.

We next evaluated the ability of our gene editing protocol to restore functional WASp expression in patient-derived primary WAS HSPCs and their progeny. The PGK-GFP reporter in the AAV6 donor vector was therefore replaced with a corrective, codon divergent and promoterless *WAS* cDNA, followed by either a synthetic Bovine Growth Hormone polyadenylation (pA) signal (W-pA) or *WAS* 3′UTR and pA signal (W-UTR). Codon optimisation introduced changes in the Cas9 target sequence of the *WAS* cDNA, in order to prevent Cas9 from re-cutting the integrated cassette, while maintaining similar levels of WASp expression as achieved with the wild-type cDNA (Supplementary Fig. 2A). Peripheral blood (PB) or bone marrow (BM) HSPCs derived from four different WAS patients (Supplementary Fig. 2B) were gene targeted using the CRISPR/Cas9 platform and each of the designed AAV6 donor vectors. To compare the proposed gene editing strategy with the existing lentiviral gene therapy for WAS^12,13^, WAS HSPCs were also transduced with a GMP-grade lentiviral vector currently used in WAS gene therapy clinical trials, which consists of a 1.6-kb fragment of the endogenous WAS promoter to drive the expression of a wild-type *WAS* cDNA (WW1.6; Fig. 2a, b). Rates of gene targeting and restoration of WASp expression were tested 5 days after editing/transduction. As detected by digital droplet PCR (ddPCR), we achieved high levels of gene marking in WAS HSPCs, with an average of 46.4% ± 8 and 24.9% ± 10.9 of knock-in when using the W-pA and W-UTR constructs, respectively (Fig. 2c). As cells derive from male WAS patients with a single X-chromosome, the frequency of monoallelic modification detected by ddPCR is a direct measure of the percentage of cells that have integrated a correct *WAS* cassette. Analysis of WASp expression by flow cytometry confirmed the results obtained by ddPCR, with an average of 45.5% ± 8.8 and 24.3% ± 6.8 of WAS HSPCs expressing the protein when targeted with the W-pA and W-UTR constructs, respectively (Fig. 2d; Supplementary Fig. 8). Following transduction with the WW1.6 lentiviral vector, 33.9% ± 13.1 of WAS HSPCs restored WASp expression with an average vector copy number (VCN) of 1 (Fig. 2e). Modified cells maintained their ability to give rise to erythroid and myeloid colonies in solid cultures, and no skewing in colony formation was observed compared to WT HSPCs and mock-electroporated WAS HSPCs (Fig. 2f).

**Fig. 3:**
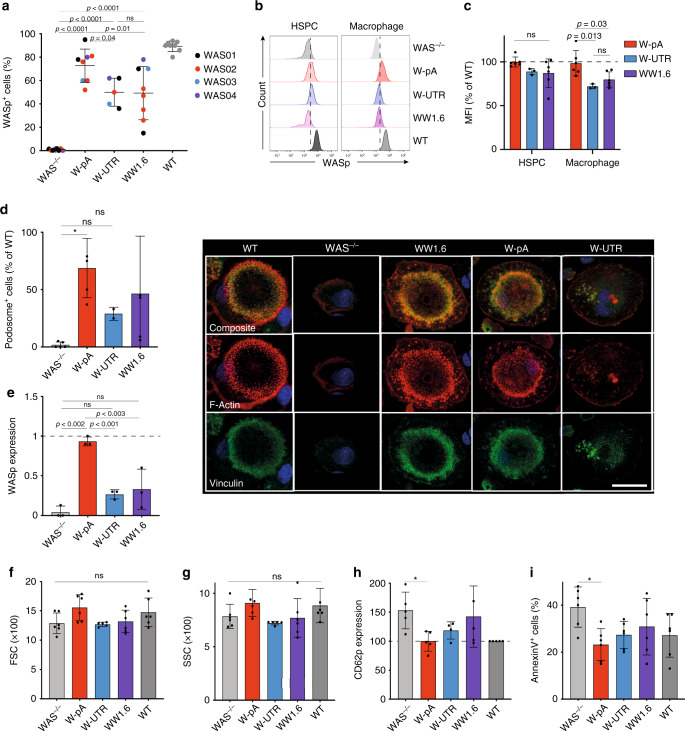
Correction of the functional defects in WAS macrophages and platelets. **a** Quantification of WASp expression in macrophages derived from mock, edited, LV-transduced WAS HSPC and wild-type (WT) HSPCs by flow cytometry (*n* = 8 independent experiments for all groups except for W-UTR (*n* = 5) using four different donor sources; exact *p*-values are shown in the panel, NS: *p* > 0.05). **b** Representative histogram overlay plot showing WASp expression in all groups, detected by flow cytometry **c** WASp expression (expressed as MFI, mean fluorescence of intensity) mediated by each of the therapeutic vector relative to that detected in WT cells. (*n* = 6 independent experiments for all groups except for W-UTR (*n* = 3) from four different donor sources; exact *p*-values are shown, NS: *p* > 0.05). **d** Left: percentage of podosome-forming macrophages for each condition relative to WT cells (*n* = 5 independent experiments for all groups except for W-UTR (*n* = 2) from four different donor sources; *p = 0.022; NS: *p* > 0.05); Right: representative images of podosomes by confocal microscopy at 63× magnification. Scale bar 10 μm. **e** Quantification of WASp expression in platelets derived from mock, edited, LV-transduced WAS HSPCs and WT HSPCs by immunoblotting (*n* = 3 experiments from three different donors; exact *p*-values are shown in the panel; NS: *p* > 0.05). **f** Size of platelets for each group described by their forward scatter (FSC) detected by flow cytometry (*n* = 6 independent experiments from four different donor sources; NS: *p* > 0.05). **g** Granularity of platelets for each group, described by their side scatter (SSC) detected by flow cytometry (*n* = 6 independent experiments from four different donors; NS: *p* > 0.05). **h** Activation profile of platelets for each group, expressed as the MFI of CD62p normalised by CD61 expression. We assigned to the unstimulated WT cells a value of 100 and normalised the other conditions accordingly (*n* = 5 experiments from four different donors; *p = 0.037, all other comparisons = *p* > 0.05). **i** Apoptotic profile of platelets (percentage of Annexin V-positive cells) for each group (*n* = 6 experiments from four different donors; *p = 0.014, all other comparisons = *p* > 0.05). Data in Fig. 3 are presented as mean ± SD. *P*-values were calculated using one-way ANOVA with Tukeys’s comparison test. Source data are provided as a Source Data file.

Macrophages represent an ideal model to study WASp reconstitution, as the absence of the protein in these cells abrogates podosome assembly^21–23^. To this end, manipulated and unmanipulated WAS HSPCs were differentiated in vitro into macrophages. As observed in WAS patients and WAS mouse models^24,25^, differentiation into CD14+ monocytes and more mature macrophages was not affected by WASp deficiency (Supplementary Fig. 3A, B). The frequency of corrected cells increased dramatically during cell differentiation, with an average of 75% ± 14, 50% ± 12 and 50.6% ± 26 of WASp-expressing macrophages derived from HSPCs modified with W-pA, W-UTR and WW1.6, respectively (Fig. 3a; Supplementary Fig. 8). Comparison of WASp mean fluorescence intensity (MFI) in cells modified with the three different constructs showed that knock-in of the W-pA cassettes led to the expression of WASp at levels comparable to those observed in WT cells, at both the HSPC and the macrophage stage (average of 100 ± 5.6% and 98.5 ± 14% of WT expression, respectively). Cells edited with the W-UTR construct or transduced with the lentiviral WW1.6 construct expressed WASp at slightly reduced levels compared to W-pA-edited cells, especially in differentiated macrophages (72 ± 2.6% and 79.8 ± 8.8% of WT expression, respectively; Fig. 3b, c). To demonstrate functional correction of the morphologic and functional defects upon integration of a normal *WAS* cDNA, we next evaluated the formation of podosomes in macrophages derived from modified WAS HSPCs by confocal microscopy. Staining of the actin cytoskeleton in fibronectin-adhering macrophages showed restored podosome assembly in all treated groups, with macrophages derived from W-pA-edited WAS HSPCs yielding the highest number of podosome-forming cells (average of 69 ± 25% compared to WT). Podosomes were found to be correctly grouped at the leading edge of polarised cells, with minimal numbers of macrophages displaying an abnormal elongated shape as seen in WASp-deficient cells (Fig. 3d, Supplementary Fig. 3C).

The fundamental pathogenesis of platelet defects in WAS is still largely elusive, but both ineffective platelet production and their accelerated elimination in periphery have been implicated^26–28^. The former hypothesis has been confirmed by recent studies highlighting the presence of platelet-intrinsic defects that may accelerate their clearance^29,30^, such as a reduced size and granularity, dysregulated activation at steady state (high CD62P expression) and increased phosphatidylserine exposure which in turn may lead to increased platelet phagocytosis. To determine the ability of our gene editing strategy to correct the abnormalities observed in this cell lineage, modified WAS HSPCs were also differentiated into platelets and functional correction was tested at this stage. As reported by others^30^, we did not observe any significant difference in the development of megakaryocytic progenitors and in the output numbers of platelets between WT and WAS HSPCs in vitro, as well as edited or lentivirally transduced WAS HSPCs (Supplementary Fig. 4A–E). Rates of targeted integration in megakaryocytic progenitors were similar to those detected in WAS HSPCs, indicating stable integration of the therapeutic cassette and absence of negative selection of edited cells. Platelets derived from W-pA edited WAS HSPCs expressed WASp at levels comparable to their wild-type counterpart, while much weaker expression was observed for both W-UTR edited and WW1.6 transduced cells (Fig. 3e, Supplementary Fig. 5F). Although this did not reach statistical significance, WAS HSPC in vitro-derived platelets displayed decreased size and granularity compared to healthy controls and knock-in of the W-pA cassette in WAS cells rescued the phenotype (Fig. 3f, g; Supplementary Fig. 9). In terms of functional changes, there was a statistically significant increased level of phosphatidylserine exposure (Annexin V) and CD62P expression in WAS platelets compared to those derived from WT HSPCs upon activation; in both cases, gene editing using the W-pA, and to a minor extent the W-UTR, AAV6 donor construct restored normal WASp levels, while WW1.6 transduced cells exhibited partial correction of the defects (Fig. 3h, i).

Overall, these results showed that a gene editing platform enables efficient knock-in of a correct WAS cDNA in up to 60% of patient-derived HSPCs, allowing physiological levels of WASp expression upon integration of the therapeutic cassette. Moreover, gene edited HSPCs are able to differentiate into mature macrophages and platelets with restored functionality.

### Editing of WAS T cells corrects their functional defects

Absence of WASp in T cells is a fundamental contributor to the development of immunodeficiency in WAS patients^31^, therefore restoration of protein expression in these cells and correction of their functional defects is mandatory for any successful therapeutic approach. In T-cells, WASp plays a crucial role in regulating actin dynamics following T-cell receptor (TCR) engagement and is involved in T-cell activation. As a result, lack of WASp leads to severe defects in the ability of T-cells to proliferate and to produce IL-2 in response to TCR stimulation^32,33^.

**Fig. 4:**
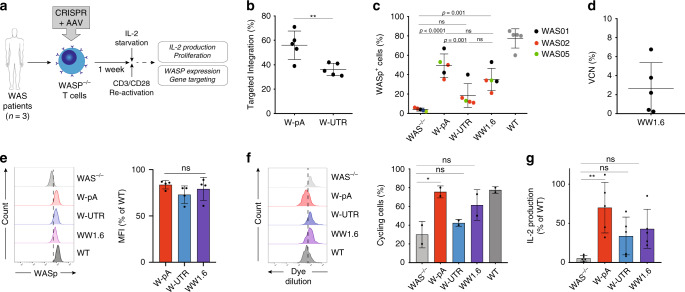
Correction of the functional defects in WAS T-cells. **a** Experimental plan to assess functional correction of the WAS defect in T-cells derived from WAS patients.This figure was created using Servier Medical Art templates, which are licensed under a Creative Commons Atrtribution 3.0 Unported License; https://smart.servier.com. **b** Rates of targeted integration achieved with the two AAV6 donor vectors detected by ddPCR (*n* = 5 experiments from three different donors; Asterisks indicate *p*-values: **p = 0.082, two-tailed unpaired Student’s *t* test)). **c** Quantification of WASP expression in mock, edited, LV-transduced WAS T-cells and healthy T-cells (WT) by flow cytometry (*n* = 5 experiments from three different donors; exact *p*-values are shown in the panel; NS: *p* > 0.05; one-way ANOVA with Bonferroni’s multiple comparison test). **d** Quantification of the number of lentiviral vector copies integrated into the genome of transduced WAS T-cells (VCN, vector copy number; *n* = 5 experiments from three different donors). **e** Left: representative histogram overlay plot showing WASp expression in mock, edited, LV-transduced WAS T-cells and wild-type T-cells, detected by flow cytometry. Right: WASp expression (expressed as MFI, mean fluorescence of intensity) mediated by each of the therapeutic vector relative to that detected in wild-type (WT) cells. (*n* = 4 experiments from three different donors; NS: *p* > 0.05, as analysed by one-way ANOVA with Bonferroni’s multiple comparison test). **f** Left: representative histogram overlay plot showing cell proliferation, as measured by fluorescent dye dilution of stimulated mock, edited, LV-transduced WAS T-cells and healthy T-cells (WT). Right: percentage of cycling cells, as measured by fluorescent dye dilution of stimulated mock, edited, LV-transduced WAS T-cells and healthy T-cells (WT) (*n* = 2 experiments from two different donors; *p = 0.034; NS: *p* > 0.05; one-way ANOVA with Bonferroni’s multiple comparison test). **g** Quantification of IL-2 production by stimulated and IL-2 starved mock, edited, LV-transduced WAS T-cells and wild-type (WT) T-cells (*n* = 5 experiments from three different donors; **p < 0.0028; NS: *p* > 0.05; one-way ANOVA with Bonferroni’s multiple comparison test). Data in Fig. 5 are presented as mean ± SD. Source data are provided as a Source Data file.

To investigate whether the HDR-mediated integration of a correct *WAS* cDNA restores sufficient levels of WASp to correct T cells intrinsic defects, we delivered CRISPR/Cas9 and AAV6 reagents to CD3/CD28 activated WASp-deficient T-cells harvested from the PB of three different WAS patients. Cells were kept in culture for 1–2 weeks post editing, after which functional correction was assessed (Fig. 4a). As shown by ddPCR analysis, targeted integration of the therapeutic cassette was achieved in 56% ± 12 and 36.2% ± 5 of WAS T-cells when delivering the W-pA and W-UTR AAV6 donor templates, respectively (Fig. 4b), with 50% ± 12 and 18% ± 4 of cells expressing WASp as detected by flow cytometry (Fig. 4c; Supplementary Fig. 8). Lentiviral vector transduction restored WASp expression in 35% ± 11 of WAS T-cells, with an average VCN of 1.6% ± 1.1 (Fig. 4d). As already observed in HSPCs, integration of the WAS cDNA with a strong BGH pA signal yielded the highest level of protein expression (83.7 ± 4.5% of WT expression), followed by the WW1.6 (79 ± 12% of WT expression) and the W-UTR (73 ± 9.4% of WT expression) constructs (Fig. 4e).

To investigate T-cell correction after gene editing, we measured the ability of modified and unmodified cells to proliferate and produce IL-2 in response to TCR/CD3 stimulation. WAS T-cells showed improved proliferation ability following integration of the therapeutic *WAS* cDNA, reaching levels comparable to T-cells derived from healthy donors when the W-pA construct was delivered (75.5% ± 6 in W-pA and 77.5% ± 3.5 in WT), and slightly lower rates with the W-UTR template (42.5% ± 3.5) and WW1.6 lentiviral vector (61.5% ± 16; Fig. 4f, Supplementary Fig. 10). Restoration of cell proliferation mirrored the rates of WAS cDNA integration achieved with each vector and reflected the presence of a proliferative advantage of WASP-expressing cells over deficient ones. When testing IL-2 production by ELISA in CD3-activated WAS T-cells before and after WAS gene transfer, we confirmed a significant restoration of cytokine secretion in gene edited and lentivirally transduced cells, reaching 66.5%, 31.2% and 41.4% of WT T-cell IL-2 production when integrating the W-pA, W-UTR and WW1.6 cassettes, respectively (Fig. 4g).

Taken together, these data demonstrate that knock-in of a normal *WAS* cDNA in WAS T-cells is highly efficient and leads to functional correction of their intrinsic defects.

### Edited WAS HSPCs retain their repopulating potential in vivo

To evaluate whether corrected WAS HSPCs retain their capacity to repopulate the BM and differentiate into all the hematopoietic lineages, gene edited and lentivirally transduced WAS HSPCs were transplanted into 8-weeks-old sub-lethally irradiated immunodeficient non-obese diabetic (NOD)-SCID Il2rg^−/−^ (NSG) mice. For this set of experiments, attention was focussed on the W-pA AAV6 donor construct, given its higher performance in terms of HDR rates and WASp expression level, and compared it to the lentiviral gene addition approach (Supplementary Fig. 5A).

**Fig. 5:**
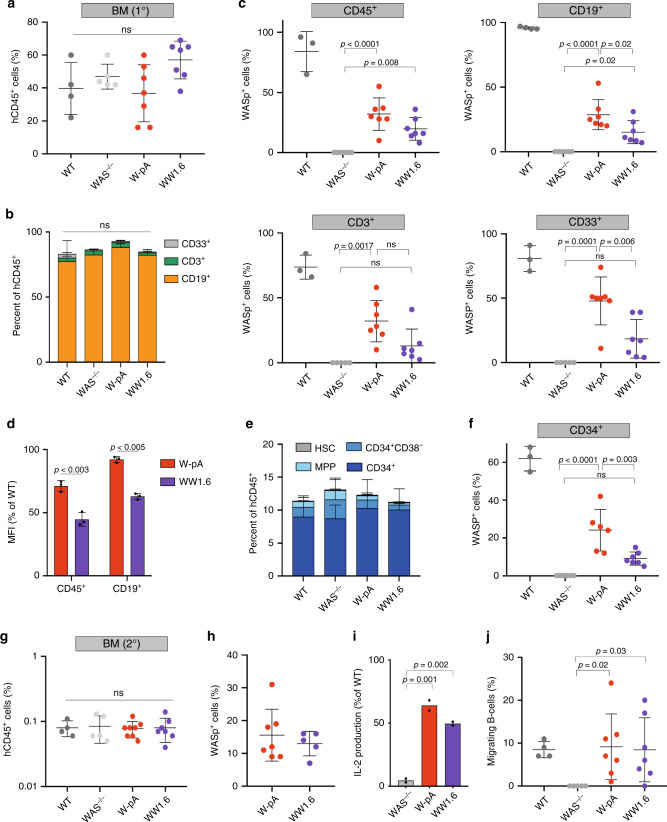
In vivo hematopoietic reconstitution by gene targeted WAS HSPCs. **a** Engraftment and **b** lineage distribution of human cells (hCD45+) in the BM of NSG mice 14 weeks after primary transplant (*n* = 7 mice for all groups except for WT (*n* = 4) and WAS−/− (*n* = 5); NS: *p* > 0.05). **c** Percentage of WASp-expressing cells in the BM for each lineage (*n* = 7 mice for all groups except for WT (*n* = 3) and WAS−/− (*n* = 5); exact *p*-values are shown in the panel). **d** WASp expression (expressed as MFI, mean fluorescence of intensity) mediated by each of the therapeutic vector in hCD45+ and CD19+ cells, relative to that detected in wild-type (WT) cells (*n* = 7 mice for all groups; exact *p*-values are shown in the panel). **e** Stem cell and progenitor distribution within BM hCD45+ human cells (*n* = 7 mice for all groups except for WT (*n* = 4) and WAS−/− (*n* = 5); for HSC, MPP and CD34+ CD38- populations: *p* = 0.018 WAS−/− versus WW1.6, *p* = 0.008 W-pA versus WW1.6. **f** Percentage of CD34+ cells expressing WASp in the BM (*n* = 7 mice for WW1.6, *n* = 6 for W-pA, *n* = 5 for WAS−/− and *n* = 3 for WT; exact *p*-values are shown in the panel). **g** Engraftment of human cells (hCD45+) in the BM of NSG mice 12 weeks after secondary transplant (*n* = 7 mice for WW1.6, *n* = 8 for W-pA, *n* = 6 for WAS−/− and *n* = 4 for WT; NS: *p* > 0.05). **h** Percentage of BM hCD45+ cells expressing WASp in secondary transplanted animals (*n* = 5 mice for WW1.6, *n* = 7 for W-pA; NS not significant). **i** Quantification of IL-2 production by mock, edited, LV-transduced thymic WAS T-cells (*n* = 2 independent experiments from pooled thymi derived from 4 to 7 mice per experimental group; exact *p*-values are shown in the panel). **j** Percentage of migrating B-cells in response to SDF-1α (*n* = 7 mice for all groups except for WT (*n* = 4) and WAS−/− (*n* = 5); exact *p*-values are shown in the panel). Data in Fig. 5 are presented as mean ± SD. *P*-values were calculated using one-way ANOVA with Bonferroni’s comparison test (**a**, **c**, **f**–**j**), two-way ANOVA with Bonferroni’s comparison test (**b**, **e**) or two-tailed unpaired Student’s *t* test (**d**). Source data are provided as a Source Data file.

Eight million HSPCs derived from one WAS patient were edited following our standard protocol or transduced with the WW1.6 lentiviral vector. A targeted integration frequency of 47.9% was observed in WAS HSPCs edited with the W-pA construct before transplantation, with 49% of cells expressing WASp as detected by flow cytometry; of the remaining cells, 44% bore NHEJ-induced indels and 7.8% were unmodified (Supplementary Fig. 5B). A VCN of 0.4 was detected in cells transduced with the lentiviral vector which, considering a 23% of transduction rate, suggested the presence of an average of more than 1.5 vector copies in transduced cells. Human engraftment was measured at week 14 after transplant and no statistically significant difference in chimerism between mock edited, edited and lentivirally transduced WAS HSPCs was observed in the BM of transplanted animals (Fig. 5a), while slightly higher numbers of human CD45^+^ cells were observed in the PB of WW1.6-transplanted animals compared to mock and gene edited ones (Supplementary Fig. 5C). Flow cytometry analysis of T-cells (CD3+), myeloid progenitors (CD33), and B-cells (CD19+) showed no differences in lineage composition in the BM and PB among the experimental groups (Fig. 5b, Supplementary Figs. 5D and6), indicating correct differentiation of manipulated WAS HSPCs. An average of 36.8 ± 15.4% of W-pA-edited human engrafted cells (hCD45+) in the BM expressed WASp, with similar levels observed in T-cells and B-cells, while CD33+ myeloid cells displayed the highest rates of gene correction (47.8% ± 20.3; Fig. 5c). Targeted integration was measured in these cells by ddPCR, with 40.7% of BM hCD45+ cells and 37% of human CD19+ splenic B-cells harbouring the therapeutic cDNA. The overall distribution of HDR-, NHEJ-corrected or unmodified cells engrafted in mice was not significantly different from that detected in the HSPC bulk population pre-transplant (Supplementary Fig. 5B), indicating absence of positive or negative selection of modified cells in vivo. Despite similar or even higher levels of human cell engraftment achieved in WW1.6 transplanted mice, the number of WASp corrected cells was generally lower compared to the gene editing group and significantly decreased in the myeloid and B-cell compartments, with a frequency of 18.5 ± 15 and 15.1 ± 9% and VCN of 0.24 and 0.33, respectively (Fig. 5c, Supplementary Fig. 5E). Our designed gene editing strategy restored semi-physiological expression of WASp in HSPC-derived progeny, reaching 71.1 ± 4.1% and 92.1 ± 2.3% of WT expression in sorted CD45+ cells and CD19+ B-cells, respectively. The reduction in WASp MFI observed in vitro in cells transduced with the lentivirus compared to gene edited cells was intensified in vivo, with only 44 ± 5.4% and 62.8 ± 2.3% of wild-type WASp expression achieved with the WW1.6 in sorted hCD45+ cells and CD19+ B-cells, respectively (Fig. 5d).

Being a life-long source of hematopoietic cells, it is of paramount importance to determine the fraction of corrected primitive stem and progenitor cells that are able to engraft the BM. Flow cytometry analysis of CD45+ cells isolated from the BM of transplanted animals showed an overall equal distribution of CD34+ cells and of more primitive populations (CD34 + CD38−, CD34 + CD38-CD90 + or HSCs, CD34 + CD38-CD90- or MPPs) among WT, WAS HSPCs, and W-pA-corrected experimental groups (Fig. 5e, Supplementary Fig. 7). In contrast, BM cells derived from the WW1.6 lentivector group, despite containing a balanced amount of CD34+ cells, showed a significantly reduced representation of HSCs, MPPs and CD34+ CD38− cells, suggesting a pronounced cell differentiation/loss of stemness upon lentiviral transduction. Moreover, while ~24.1 ± 11% of BM CD34+ cells expressed WASp in the gene editing experimental group, only 9.1 ± 2.5% of CD34+ cells were detected in the BM of animals transplanted with WW1.6-modified HSPCs that expressed WASp to a similar level (Fig. 5f). To further assess the ability of our gene editing platform to target long-term repopulating HSCs (LT-HSCs), secondary transplants were performed into sub-lethally irradiated NSG mice using hCD45+ cells harvested from the BM of primary transplanted animals (Supplementary Fig. 5A). Despite overall low level of chimerism observed in the BM of mice 12 weeks after transplant, no differences in the percentage of human cell engraftment and lineage composition were observed among the experimental groups (Fig. 5g, Supplementary Fig. 5F). WASp expression was detected in all the animals transplanted with gene edited or LV-transduced WAS HSPCs, with an average of 16 ± 7.9% and 13 ± 3.7% WASp-positive cells respectively (Fig. 5h).

Having demonstrated the successful engraftment of gene edited cells in transplanted mice, we sought to determine the functionality of mature lymphoid cells derived from corrected WAS HSPCs isolated from the transplanted mice. Human CD3+ T-cells were isolated from murine thymi 14 weeks after primary transplantation and cultured in standard conditions for a week, then restoration of IL-2 production in CD3-activated T-cells was tested. By estimating IL-2 production, the restoration of T-cell responsiveness to TCR stimulation was determined, with W-pA-edited and WW.16 reaching 64 ± 5.6% and 49.5 ± 2.1% of WT T-cell IL-2 production, respectively (Fig. 5i).

As the largest human hematopoietic cell population in NSG mice was represented by B-cells (Fig. 5b), alleviation of their intrinsic phenotypic abnormalities were evaluated. WASp-deficient B-cells are characterised by a defective actin cytoskeleton, failure to assemble filopodia, and decreased migration ability both in vitro and in vivo^25,34^. B-cells were isolated from the spleen of transplanted animals and their chemotactic response to SDF-1α was tested by transwell migration assay. As shown in Fig. 5j, while WAS-deficient cells were unable to migrate, corrected B-cells belonging to both gene editing and lentivector groups were responsive to chemotactic cues, at levels comparable to WT HSPCs.

Overall, these results showed that gene edited WAS patient-derived cells retain their ability to repopulate the BM of recipient animals and sustain normal hemopoiesis in primary and secondary transplants, further confirming the safety and feasibility of the approach. WASp expression remained stable throughout 26 weeks post-transplant, with only a slight reduction in gene targeting rates compared to the pre-transplant time point, indicating efficient modification of LT-HSCs. Furthermore, knock-in of a normal *WAS* cDNA into WAS HSPCs can correct the phenotypic and functional defects of lymphocytes derived from edited cells that engrafted the BM of immunodeficient mice.

### Off-target analysis confirms the specificity of the platform

**Fig. 6:**
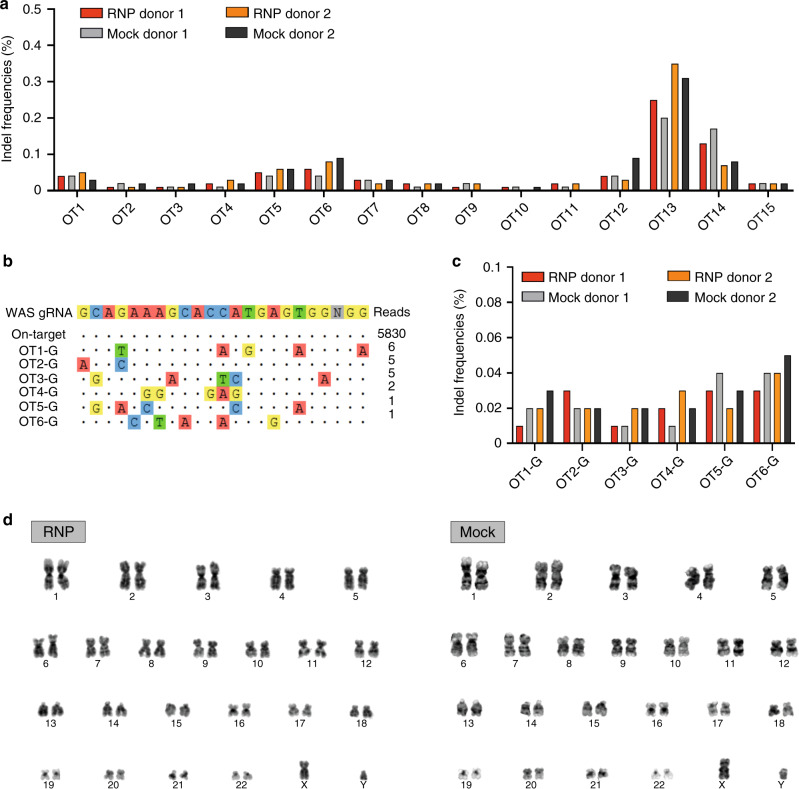
Genotoxicity analysis in edited HSPCs. **a** Targeted high-throughput sequencing of off-target sites in edited (RNP) or electroporated-only (Mock) HSPCs (*n* = 2 experiments from two different donors; no significant difference between RNP and Mock samples when pooled and analysed with two-tailed paired Student’s *t* test). **b** Sequences of off-target sites identified by GUIDE-seq for WAS gRNA. The intended target sequence is shown in the top line with mismatches to the on-target site shown and highlighted in colour. GUIDE-seq sequencing read counts are shown to the right of each site. GUIDE-seq sequencing read counts are shown to the right of each site. **c** Targeted high-throughput sequencing of off-target sites in edited (RNP) or electroporated-only (Mock) HSPCs (*n* = 2 experiments from two different donors; no significant difference between RNP and Mock samples when pooled and analysed with two-tailed paired Student’s *t* test). **d** Karyotype analysis of unmanipulated (Mock) and genome edited (RNP) HSPCs showed no chromosomal abnormalities (100 cells analysed for each condition). Source data are provided as a Source Data file.

One of the potential concerns associated with gene editing is the introduction of unwanted genetic modifications at off-target sites, posing a huge risk for clinical therapeutic applications involving engineered nucleases. To determine the specificity of our gRNA targeting *WAS*, we delivered the CRISPR/gRNA RNP to HSPCs derived from two different healthy donors and assessed the presence of indels at non-specific sites, predicted using the bioinformatic tool COSMID^35^. Off-target activity at 15 genomic sites showing high homology with WAS gRNA target sequence (up to 3 mismatches) was measured by targeted deep-sequencing in treated and mock-treated HSPCs. At a read depth of 50,000x we could not detect any genetic disruption at a statistically significant frequency compared to mock-treated controls in all of the genomic sites tested (Fig. 6a, Supplementary Fig. 11A). To further investigate the safety of our approach, we utilised an unbiased, genome-wide analysis tool and GUIDE-seq (genomewide, unbiased identification of DSBs enabled by sequencing)^36^. Analysis of the sequencing data with two different analysis softwares^37,38^ retrieved 0 and 6 different off-target sites respectively, at extremely low frequency (Fig. 6b, Supplementary Fig. 11B). However, targeted deep sequencing of the six sites identified by GUIDE-seq analysis in CRISPR-treated and mock-treated HSPCs from two different healthy donors demonstrated no significant gene disruption at the genomic locations tested (Fig. 6d).

To confirm the lack of genomic instabilities caused by the gene editing process, we performed karyotype analysis on HSPCs derived from male healthy donors. This analysis demonstrated absence of chromosomal aberrations in 100 out of 100 RNP-treated cells and in 99 out of 100 mock-electroporated cells (Fig. 6c), confirming the high specificity and the lack of genotoxicity of our gene editing platform.

## Discussion

Here, we report the successful application of a CRISPR/Cas9-based gene editing platform to correct an extremely complex primary immunodeficiency, Wiskott–Aldrich Syndrome, through editing of the *WAS* locus in primary human HSPCs. While there has been a limited number of studies showing editing of the *WAS* gene in cell lines and induced pluripotent stem cells^39,40^, this work represents the first evidence of therapeutically relevant genetic correction in healthy and patient-derived hematopoietic stem and differentiated cells.

Our strategy restores normal WASp expression by integrating a full-length *WAS* cDNA next to *WAS* endogenous translational start site on the X chromosome, utilising a highly-specific gRNA targeting *WAS* 5′UTR and an AAV6 donor vector carrying the therapeutic cassette in a promoterless configuration. This approach brings with it several benefits, including: (a) targeted integration of one correct copy of *WAS* per cell, maintaining a normal gene copy number; (b) transcriptional regulation by endogenous regulatory elements, ensuring physiological *WAS* expression upon integration of the cassette; (c) correction of all disease-causing mutations with one set of reagents, therefore representing a universal platform that could be applied to all WAS patients. Through systematic optimisation of the gene editing protocol and cell culture conditions, including to clinical scale, we achieved extremely high levels of targeted integration, with a frequency of knock-in reaching up to 69 and 61% when delivering a PGK-GFP reporter cassette in healthy human HSPCs or the therapeutic *WAS* cDNA in patient-derived HSPCs, respectively. Insertion of one normal copy of the gene in WAS HSPCs resulted in the correction of WAS typical morphological and functional defects in mature hematopoietic cells – macrophages, platelets, T-cells and B-cells – derived from edited stem cells in vitro, and consistently across different experiments and HSPC donor sources. Full correction of WAS cell phenotypes resulted from the restoration of near physiological levels of WASp expression which was achieved in particular by utilising a strong BGH poly-A signal, while expression using the *WAS* endogenous 3′UTR and poly-A signal was consistently much lower; this may reflect the need of additional endogenous sequences flanking the 3′UTR region in the WAS locus – such as those contained in the 3′ introns^41^ — to recapitulate normal WASp expression. Inclusion of such sequences in the donor vector may however increase the size of the integrant and therefore be detrimental to the rates of targeted integration of the therapeutic cassette, as already observed for the W-UTR construct. Nevertheless, integration of the W-pA cassette containing a synthetic poly-A signal did not alter WASp expression and sustained normal protein levels.

Besides achieving a stable and regulated expression of the therapeutic gene, a genome editing therapeutic approach must correct a sufficient amount of LT-HSCs to be considered a viable and potentially life-long treatment for genetic blood disorders. We demonstrated successful integration of a PGK-GFP reporter cassette in >60% of FACS-sorted primitive stem cells and multipotent progenitors while preserving high cell viability and repopulating ability. Successful gene correction of patient-derived LT-HSCs by delivery of the therapeutic donor vector resulted in functional and stable restoration of WASp expression in myeloid and lymphoid progenies in NSG mice up to 26 weeks after transplant, with an average of 16% of targeted LT-HSCs as determined in secondary transplantation studies. Although a reduction in the percentage of corrected cells was observed after primary and secondary transplantation, the rate of decrease was much lower (<10% in primary transplant and ∼50% in secondary transplant) compared to what has been reported in other studies using CRISPR-Cas9 to edit HSPCs^15,16,42^; moreover, the overall percentage of HDR-corrected cells detected after secondary transplantation is the highest reported so far in patient-derived HSPCs. The high rates of human cell engraftment observed in the BM and PB of NSG mice transplanted with edited patient-derived HSPCs, together with the generation and maintenance of corrected differentiated cells in vitro and in vivo, confirm the lack of toxicity of the editing procedure in LT-HSCs.

Lentiviral gene therapy using autologous HSPCs has been recently shown to be a valuable therapeutic option, providing limited toxicity and substantial clinical improvement^12,13^. When following clinical transduction protocols, the WW1.6 lentiviral vector achieved consistently lower levels of gene correction compared to the W-pA construct in mature cells, despite an average number of integrated vector copies similar (in HSPCs) or even higher (in T-cells) than that achieved through gene editing. This is reflected by the overall lower level of WASp expression observed in several hematopoietic lineages analysed in vitro and in vivo; while WASP MFI in lentivirally transduced WAS T-cells was almost equivalent to that observed in gene edited cells, a more pronounced difference was visible in mature macrophages and B-cells, especially in vivo. Our data mirror the generally lower levels of WASp expression within the B-lymphocyte and myeloid compartment compared to T-cells observed in WAS patients treated with the WW1.6 lentiviral vector, and confirm the results obtained in previous clinical^11^ and pre-clinical studies^43^. These findings suggest that additional *WAS* regulatory regions located outside of the 1.6-kb-long promoter region may be required to fully restore WASp expression. As incomplete WASp reconstitution in B- and myeloid cells has been linked to persistence of inflammatory complications and autoimmunity^12,13,43^, reliable and robust WASp restoration through a gene editing platform could potentially reduce such risks. Full reconstitution of WASp expression could also contribute to more reliable correction of thrombocytopenia, and therefore to application of this approach to patients with attenuated WAS who predominantly manifest thrombocytopenia and bleeding risk^12,13,29^. Although limited to an in vitro platelet differentiation system, our data suggest that targeted integration of the W-pA cassette can restore normal levels of WASp in megakaryocyte progenitors and mature platelets, correcting the platelet-intrinsic defects observed in WAS patients. Due to the lack of a reliable animal model that recapitulates a WAS platelet phenotype^44,45^ while allowing efficient human cell engraftment, complete correction of thrombocytopenia can be ultimately confirmed only in patients.

The controlled transgene integration achieved with our CRISPR/Cas9 platform provides a potentially safer strategy of gene correction, overcoming the risk of oncogenic transformation and genotoxicity associated with the semi-random integration pattern of viral vectors^46^. This is particularly important in the case of WAS, where patients are naturally predisposed to lymphoid malignancy^47^. While indels generation at off-target sites still poses a risk to the use of engineered nucleases, we demonstrated absence of non-specific targeting of our CRISPR/Cas9 reagents and of major chromosomal rearrangements using biased and unbiased off-target detection tools. Even though we could have missed additional mutations introduced by the nuclease, the lack of toxicity, of abnormal clonal expansion and of oncogenic transformation in animals infused with gene edited WAS HSPCs 26 weeks after transplantation support the overall safety of our approach. Nevertheless, further work to verify absence of genotoxicity in more depth will be required to validate the translation of our gene editing platform from bench to bedside.

In conclusion, our study provides a clear demonstration of the efficacy and potential safety of a CRISPR-based gene editing approach to treat WAS. This strategy could provide a valuable therapeutic alternative for all patients affected by this disease and could enable the translation of such technology to a much wider range of HSC blood disorders.

## Methods

### Statistical analysis

All the experiments were carried out at least thrice independently. All data are presented as means ± SD. Animals of similar age, weight and sex were grouped randomly, and the number of animals per group was 4–8. Statistical analyses were conducted using GraphPad Prism 7 (GraphPad Software Inc, USA). Specifics of each statistical test used can be found in Figure legends. *P* < 0.05 was considered significant for all tests.

### Selection of gRNA

Three different gRNA (#1, #2 and #3) targeting *WAS* exon 1, each with 20 nucleotide length sequences, were identified using the online design tool created by the Zhang lab (https://zlab.bio/guide-design-resources). Chemically modified gRNA#1 (Synthego, USA) contained 2-O-methyl-3′-phosphorothioate at the three terminal positions at both 5′ and 3′ ends.

### Cloning of donor templates and AAV6 production

All homology based donor template of Adeno-Associated Virus type 6 (AAV6) vector were cloned into pAAV-MCS plasmid containing AAV2 specific ITRs. The left and right homology arms for AAV6_PGK-GFP and therapeutic AAV6 donors (coW-pA and coW-UTR) were 722 bp and 758 bp in length, respectively. Both AAV6 donor vectors contain a codon-optimised 1.5-kb-long *WAS* cDNA (co*WAS*), which was designed to contain silent mutations that generated 80% sequence homology to the endogenous, wild-type gene. In the coW-pA donor construct, the co*WAS* cDNA is followed by a Bovine Growth Hormone (BGH) polyA tail PCR-amplified from the pX458 plasmid (Addgene plasmid #48138). In the coW-UTR donor vector, a 270-bp fragment corresponding to the 3′UTR region, which comprises a polyadenylation signal, of human *WAS* gene was cloned after the stop codon of co*WAS* cDNA. Production of AAV6 was carried out by transfection of 293T cells with the donor template plasmid and pDGM6 Rep/Cap helper plasmid. After 72 h of transfection, both supernatant and pellet were harvested and combined together before purification using iodixanol gradient. AAV6 particles were extracted at the 60-40% iodixanol gradient and dialysed three times in 1× PBS (ThermoFisher Scientific, USA) with 5% sorbitol (Sigma-Aldrich, UK) in the final step using a 10K MWCO Slide-A-Lyzer G2 Dialysis Cassette (ThermoFisher Scientific). The vector was titrated using Quick Titre AAV quantification kit (Cell BioLabs, USA), aliquoted and stored at −80 °C until use.

### Human CD34^+^ HSPCs culture

Mobilised peripheral blood from healthy donors and WAS patients were isolated under written informed consent. Within 24 h of scheduled apheresis, CD34^***+***^ haematopoietic stem and progenitor cells (HSPCs) were purified using CD34+ Microbead kit (Miltenyi Biotec, UK) according to the manufacturer protocol. Purity was assessed by FACS staining with anti-human CD34 BV 421 antibody (clone 561, BioLegend, USA). For long-term storage, the cells were frozen in CryoStor cell cryopreservation media (Sigma-Aldrich). After thawing cells were cultured in StemSpan ACF (StemCell Technologies, USA) supplemented with SCF (100 ng/ml, Peprotech, UK), TPO (100 ng/ml, Peprotech), FLT3-ligand (100 ng/ml, Peprotech) and IL-3 (60 ng/ml, Peprotech, UK). Cells were incubated at 37 °C/5% CO_2_ for 2 days prior to electroporation.

### Human primary T-cell culture

T cells from peripheral blood of healthy donors or WAS patients were obtained using Ficoll density gradient under written informed consent. After centrifugation at 400 × *g* for 30 min without brake, mononuclear cells were carefully isolated and cultured in X-Vivo 15 (Lonza, UK) supplemented with human AB serum (5%, BioIVT, UK) and IL-2 (100 IU/ml, Peprotech) and stimulated with human CD3-CD28 T-Activator dynabeads (ThermoFisher Scientific) for 3 days prior to electroporation.

### Electroporation and transduction

After 2 days in culture, CD34^+^ HSPCs were electroporated using Neon Transfection kit (ThermoFisher Scientific). Briefly, 0.2 × 10^6^ cells were centrifuged and re-suspended in ribonucleoprotein (RNP) complex. The RNP was made by incubating gRNA and High Fidelity Cas9 protein (Integrative DNA Technologies, USA) at a molar ratio of 1:2 at 37 °C for 15 min. The condition for electroporation was 1600V, 10 ms, three pulses. For scale-up experiments, 1.25 millions, 5 millions or 20 millions HSPCs were electroporated in OC-25, OC-100 or OC-400 cuvettes using a MaxCyte CTX Flow electroporator (MaxCyte, USA). Following electroporation, cells were seeded at concentration of 1 × 10^6^ cells per ml and incubated at 37 °C for 15 min after which AAV6 at 50,000 MOI (Vector genomes/cell) was added and incubated. For T cells, after 72 h of activation, the activation beads were removed using DynaMag-15 magnetic separator (ThermoFisher Scientific) and edited following the above protocol. CD34^+^ HSPCs or T cells were in parallel transduced with a GMP grade WW1.6 lentiviral vector produced by Genethon, France. Transduction was performed in two rounds, each at an MOI of 100 for 12 hours.

### Editing of CD34^+^ HSPCs subsets

At day 0, CD34^+^ HSPCs were thawed and stained with the following antibody panel: CD34 BV 421, CD38 APC-Cy7 (clone HIT2, BioLegend), CD90 PE-Cy7 (clone 5E10, BioLegend) and CD45RA APC (clone HI100, BioLegend). The cells were then FACS-sorted into different subsets including CD38+ cells, haematopoietic stem cells (HSCs) and multipotent progenitors (MPPs). Unsorted cells were taken as an experimental control. After 2 days, both sorted and unsorted cells were electroporated and transduced with AAV6_PGK-GFP. At days 5  and 12  post editing, the cells were stained with the above antibody panels to determine the percentage of HSCs and MPPs as well as evaluate GFP expression by FACS. Cell yields were calculated by adding 10 microliters of Count Bright Absolute Counting Beads (ThermoFisher Scientific), following the manufacturer’s protocol.

### Differentiation of CD34^+^ HSPCs into macrophages in vitro

After 4 days of editing with the therapeutic AAV6 donors, WAS CD34^+^ HSPCs were seeded at a density of 40,000 cells per cm^2^ in IMDM (ThermoFisher Scientific) supplemented with SCF (20 ng/ml), FLT3-ligand (30 ng/ml), IL-3 (30 ng/ml), M-CSF (30 ng/ml, ThermoFisher Scientific) and FCS (20%, ThermoFisher Scientific) at 37 °C/5% CO_2_. After 7 days in culture, the cells were isolated for CD14+ monocytes using CD14+ Microbead kit (Miltenyi Biotec) according to the manufacturer’s protocol. Purified CD14+ monocytes were cultured in 24-well plate containing fibronectin (Bio-Techne, UK) pre-coated coverslip for 7 days in macrophage differentiation medium after which podosome formation was assessed via confocal microscopy (Inverted Zeiss LSM 710 confocal microscope, Zeiss). The macrophage medium comprised of RPMI (ThermoFisher Scientific) supplemented with FCS (10%), Penicillin-Streptomycin-Glutamine (1×) and M-CSF (50 ng/ml). In parallel, cells not used for confocal microscopy were seeded in non-coated 24-well plates for further 7 days in macrophage differentiation medium to detect WASp expression. Correct differentiation of macrophages was assessed by staining with anti-human CD14 PerCP Cy5.5 (clone G1D3, ThermoFisher Scientific) and anti-human CD16 APC (clone GB16, ThermoFisher Scientific) antibodies.

### Differentiation of CD34^+^ HSPCs into platelets in vitro

Platelet differentiation protocol was adapted from Perdomo et al.^48^. Briefly, day 4 post-editing WAS CD34^+^ HSPCs were seeded at a density of 1 × 10^6^ cells per ml in StemSpan ACF medium supplemented with TPO (50 ng/ml) at 37 °C/5% CO_2_. After 9 days, differentiation of megakaryocytes was evaluated by staining with anti-human CD41a FITC (clone HIP8, BioLegend) and anti-human CD42b APC (clone HIP8, BD Bioscience, UK) antibodies before FACS analysis. On the same day, 10,000 cells were cultured in 48-well plate containing 200 µl of the same above medium and cultured for further 10 days after which various tests including platelet differentiation, phenotype (size and granularity) and functionality (CD62p activation and Annexin V assay) were assessed. FACS staining with anti-human CD61 APC (clone VI-PL2, BioLegend) was used to confirm platelet differentiation. Within the CD61+ population, the size and granularity of platelets were determined from FSC and SSC values respectively. The ratio of CD62p/CD61 mean fluorescence intensity (MFI) was used to normalise the expression of activated platelets with healthy wild-type control being assigned a value of 100. To assess apoptosis, activated platelets were stained with 5 µl of APC conjugated Annexin V antibody (BD Bioscience) in the presence of 1× binding buffer. After incubation for 15 min, the reaction was quenched by the addition of 400 µl of 1× binding buffer before analysing the percentage of Annexin V + platelets by FACS.

### Methylcellulose CFU assay

The colony-forming unit (CFU) assay was performed by seeding 500 cells in six-well plates containing MethoCult Enriched (StemCell Technologies) after 4 days of editing. After 14 days of incubation at 37 °C/5% CO_2_, different types of colonies including CFU-Erythroid (E), CFU-Macrophage (M), CFU-Granulocyte (G), CFU-GM and CFU-GEM were counted based on their morphological appearance.

### Measuring INDEL frequencies

INDEL frequencies were quantified using the TIDE software (http://shinyapps.datacurators.nl/tide/) and targeted deep sequencing. Briefly, genomic DNA was extracted 4 days post editing using DNeasy Blood and Tissue extraction kit (Qiagen, UK). After isolation, the regions of interest were PCR amplified and sequenced. The primers and probes sequences are detailed in Supplementary Table 2. Amplicons were subjected to end repair, adaptor ligation and an indexing PCR using the NEBNext® Ultra™ II DNA library prep kit for Illumina (New England BioLabs, UK) according to the manufacturer’s instructions. The denatured amplicons were loaded at 12 pM into the Illumina MiSeq Reagent Kit V2 - 500 cycle according to the manufacturer’s instructions. INDEL formation was evaluated using CRISPResso^49^.

### Detection of WASp expression

Detection of intracellular WASp in CD34^+^, monocytes, macrophages and T cells was carried out by fixing the cells with 4% paraformaldehyde (PFA) and followed by incubation with primary mouse anti-human WASP antibody (1 in 100 dilution; Clone 5A5, BD Bioscience) in 0.1% Triton X-100. The cells were stained with secondary goat anti-mouse IgG Alexa Fluor 647 antibody (1 in 200 dilution; clone Poly4053, BioLegend) to determine percentage of WASp-positive cells by FACS. To check WASp expression within human CD45+ lineage and stem cell populations, primary rabbit anti-human WASp antibody (1 in 100 dilution; clone EP2541Y, Abcam) and secondary donkey anti-rabbit IgG Alexa Fluor 647 antibody [1 in 100 dilution] (clone Poly4064, BioLegend) were used. For CD34^+^ derived platelets, western blot was used to detect expression of WASp. Briefly, 20 µg of protein extracts from platelets were separated using NuPAGE 4-12% Bis-Tris gel (ThermoFisher Scientific) and transferred onto PVDF membrane. The protein was probed with primary mouse anti-human WASp antibody (1 in 500 dilution) followed by incubation with HRP conjugated sheep anti-mouse IgG antibody (ThermoFisher Scientific). The expected 68 kDa WASp band on the membrane was visualised using GeneGnome chemiluminiscence imaging system (SynGene, UK). WASp expression were quantified using ImageJ software. Briefly, a rectangular selection was made around WASp band to determine the intensity. The corresponding intensity was then normalised to the intensity of GAPDH loading control and to WASp expression in WT samples.

### Digital Droplet PCR analysis

Digital Droplet PCR (ddPCR) was performed to measure the frequency of codon-optimised *WAS* cDNA integration in the edited samples. Extraction of gDNA was carried out as mentioned above. In a total volume of 22 µl ddPCR, 20 ng of genomic DNA was combined with 10 µM each of target primer and FAM probe mix, 10 µM each of reference primer and HEX probe mix, 1× ddPCR Supermix probe without dUTP (Bio-Rad, UK) and nuclease-free water. The primers and probes sequences are detailed in Supplementary Table 2. The individual droplets were generated using QX100 Droplet Generator (Bio-Rad) and subsequently amplified in a Bio-Rad PCR thermocycler. The optimised amplification steps were: step 1 – 95 °C for 10 min; step 2 (49 cycles) – 94 °C for 1 min, 60 °C for 30 s, 72 °C for 2 min; and step 3 – 98 °C for 10 min. The Droplet Reader and QuantaSoft Software (both from Bio-Rad) were used to record and analyse the positive and negative fluorescence droplets according to the manufacturer’s guidelines (Bio-Rad). The percentage of integration was calculated as the ratio of FAM to HEX signal after normalisation against the reference signal.

### Validation of predicted off-target sites by NGS

Potential off target sites for *WAS* gRNA #1 in the human genome were identified using the web tool COSMID^35^. After off-target site ranking, 15 sites were selected for off-target screening. Frozen male peripheral blood derived CD34+ HSPCs were cultured for 2 days prior to electroporation with RNP complex as described above. After 72 h post RNP electroporation, genomic DNA was extracted and PCR was performed using the above top 15  off target primers. PCR amplicons on mock electroporated cells were taken as negative untreated control. Forward and reverse off target primers were designed around the predicted off-target sites in order to amplify fragments of 200 bp. The PCR-purified amplicons were subjected to end repair, adaptor ligation and an indexing PCR using the NEBNext® Ultra™ II DNA library prep kit for Illumina (New England BioLabs) according to the manufacturer’s instructions. The denatured amplicons were loaded at 12 pM into the Illumina MiSeq Reagent Kit V2 - 500 cycle according to the manufacturer’s instruction. The FASTQ files were analysed for indels using the command line version of CRISPResso^49^ considering 40 bp around the supposed cleavage site and not taking into account substitutions to reduce background signals. The derived INDEL proportions of the treated samples were statistically compared to the corresponding untreated values in a one-sided *Z*-test. For this purpose, the RNP treated values were corrected by the standard deviation of the untreated samples in order to account for the variability of the measurements.

### GUIDE-seq

Identification of potential off-target sites by GUIDE-seq was performed by Creative Biogene. One million HEK293T cells were transfected with 12 µg HIFI Cas9, 4 µg *WAS* gRNA #1 and 5 pmol of dsODN using Lonza Nucleofector 4-D (program CM-137). At 48 h post transfection, genomic DNA was extracted and sheared using a Covaris S220 Focussed-ultrasonicator to an average length of 500 bp. After end-repaired, A-tailed and ligation with adaptors containing 8-nt random molecular index, the DNA library was sequenced using Illumina Miseq. The subsequent datasets were analysed using either the *guideseq* Python package software^37^ or the *GUIDEseq* Bioconductor package software^38^.

### ELISA assay

T cells were harvested 7 days post editing and 100,000 cells were seeded on 96-well plate at a concentration of 1 × 10^6^ cells per ml. Cells were cultured in X-Vivo 15 medium supplemented with human AB serum (5%) and stimulated with human CD3-CD28 T-Activator Dynabeads for 24 h at 37 °C/5% CO_2_. The supernatant was processed for IL-2 production by ELISA assay kit (ThermoFisher Scientific) according to manufacturer instruction.

### T-cell proliferation assay

Seven days after editing, T cells were labelled with 5 µM of Cell Trace Violet dye (ThermoFisher Scientific) according to manufacturer’s instructions. Labelled cells were seeded on a 96-well plate pre-coated with 1 µg/ml of anti-CD3 antibody (clone OKT3, BioLegend) and cultured in X-Vivo 15 medium supplemented with human AB serum (5%). After 3 days, cellular proliferation was assessed by dye dilution by FACS analysis. Samples stained at day 0 were used as a negative control.

### Transplantation of genome edited CD34^+^ HSPC in NSG mice

NSG adult female mice (6–8 weeks old) were purchased from Charles River and were sub-lethally irradiated (3 Gy) 24 h prior to transplantation. WAS CD34^+^ HSPCs were thawed and cultured. At day 2 after editing, 0.5 × 10^6^ viable cells were injected via tail vein into mice with a 27 gauge × 0.5 inch needle. After 8 and 14 weeks post transplantation, mice peripheral blood from tail vein was lysed with 1× RBC lysis buffer (ThermoFisher Scientific) and stained with anti-human CD45 APC antibody (clone HI30, BioLegend) to evaluate human CD45+ engraftment by FACS. At week 14 post transplantation, level of human engraftment and lineage composition were determined in the bone marrow and peripheral blood. Briefly, bone marrow cells were harvested by flushing tibiae and femurs with 1× PBS and passing through 40 µm strainer. Mononuclear cells were blocked with Fc blocking solution (BioLegend) and stained with the following antibody panels; CD45 BV421, CD19 PerCp Cy5.5 (clone HIB19, BioLegend), CD33 FITC (clone P67.6, BD Bioscience) and CD3 PE (clone OKT3, BioLegend) for FACS analysis. To determine human stem cell composition within bone marrow, cells were stained with the following antibody panels; CD45 BV421, CD34 FITC, CD38 APC-Cy7, CD90 PE-Cy7 and CD45RA PerCP Cy5.5. Thymi were also harvested and grinded against 40 µm strainer. The resulting cell suspension were cultured in X-Vivo 15 supplemented with human AB serum (5%) and IL-2 (100 IU/ml) before stimulating with human CD3-CD28 T-Activator Dynabeads. After 72 h, isolated human T cells were processed for ELISA assay as described above. For secondary transplantation, the bone marrow cells were pooled together from each mice cohort and human CD45 isolation was performed using CD45+ microbead kit (Miltenyi Biotec) according to the manufacturer’s protocol. A minimum of 0.5 × 10^6^ CD45+ cells were injected via tail vein into 8 h sub-lethally irradiated (3 Gy) 6–8-weeks-old NSG female mice. The level of human CD45+ engraftment was determined from peripheral blood and bone marrow at 8 and 12 weeks post-secondary transplantation, respectively and analysed by FACS as described above.

### Chemotaxis assay

Human B-cells were selected from murine spleens using CD20+ microbead kit (Miltenyi Biotec) according to the manufacturer’s instructions. Purified B-cells were cultured overnight at 37 °C/5% CO_2_ in complete RPMI medium (FCS [10%], Penicillin-Streptomycin-Glutamine [1×]). In the next day, 50,000 viable cells were re-suspended in 100 µl of complete RPMI and seeded on 5 µM pore size Transwell inserts (Sigma-Aldrich). The lower chamber of the 24-well plate was filled with 600 µl of complete RPMI medium supplemented with human SDF-1α (250 ng/ml, Peprotech). After 37 °C/5% CO_2_ incubation for 3 h, the transmigrated cells collected in the lower chamber were stained with anti-human CD19 PerCp Cy5.5 antibody and analysed by FACS. The percentage of migration was estimated as (total number of viable CD19+ cell in the lower chamber)/(total number of initial cell in the upper chamber) × 100.

### Confocal microscopy

After culturing CD14+-derived macrophages for 7 days in fibronectin coated coverslip, cells were washed with 1× PBS. This was followed by 4% PFA fixation for 20 min and permeabilisation with 0.1% Triton X-100 for 5 min. Non-specific binding was blocked with 1× PBS containing 5% Bovine Serum Albumin for 1 h at room temperature. To detect podosomes, samples were first stained with primary mouse anti-human vinculin antibody (1 in 500 dilution; clone V4505, Sigma-Aldrich) for 1 h at room temperature. After washing three times with 1× PBS, incubation with secondary goat anti-mouse IgG Alexa Fluor 488 (1 in 500 dilution; clone H + L, ThermoFisher Scientific) was carried out for further 45 min at room temperature. Within the same period, the cells were stained with Alexa Fluor 635 phalloidin (1 in 200 dilution; ThermoFisher Scientific). Fluorescent microscopic images were taken with LSM 710 Zeiss confocal microscope using Zen 2009 light edition software (Zeiss, UK). For each experiment, 50–100 cells per sample from at least three fields of view were counted to assess podosome formation.

### FACS analysis

BD FACSAria II (BD Bioscience) instrument was used for cell sorting of CD34^+^ HSPCs. For all flow cytometry analysis, a BD LSRII instrument (BD Bioscience) was used. Antibody dilutions used for FACS staining was 1 in 100. For data anlaysis, FlowJo v10 software (FlowJo LLC, USA) was used.

### Karyotype analysis

Healthy mobilised peripheral blood derived CD34^+^ HSPCs were electroporated as described above. After 4 days in culture, 10 × 10^6^ cells from mock and RNP treated cells were processed by Clinical Cytogenetics Lab at Great Ormond Street Children Hospital. Karyotype analysis was performed on 100 cells from each condition.

### Ethics and animal approval statement

For usage of human CD34^+^ HSPC from healthy and WAS donors, informed written consent was obtained in accordance with the Declaration of Helsinki and ethical approval from the Great Ormond Street Hospital for Children NHS Foundation Trust and the Institute of Child Health Research Ethics (08/H0713/87).

For experiments involving animals, mice were housed in a 12-h day-night cycle with controlled temperature and humidity. The ventilated cages had sterile bedding and everyday supply of sterile food and water in the animal barrier facility at University College London.

Mice were bred and maintained in accordance with UK Home Office regulations, and experiments were conducted after approval by the University College London Animal Welfare and Ethical Review Body (project license 70/8241).

### Reporting summary

Further information on research design is available in the Nature Research Reporting Summary linked to this article.

## Supplementary information

Supplementary Information

Reporting Summary

## Data Availability

Sequencing data have been deposited at [Bioproject ID PRJNA637588] under accession code PRJNA637588 (https://www.ncbi.nlm.nih.gov/bioproject/637588). We declare that the data supporting the findings of this study are available within the paper and its Supplementary Information Files or from the authors upon request. The source data underlying plots in Figs. 1c–g, j, k, l, 2c–f, 3a, c–i, 4b–g, 5a–j and 6a, c, and Supplementary Figs. 1b, c, f, g, 3b, 4b, c, f and 5b–f are provided as a Source Data file. Source data are provided with this paper.

## Source data

Source data

## References

[1] Worth AJ, Thrasher AJ (2015). Current and emerging treatment options for Wiskott-Aldrich syndrome. Expert Rev. Clin. Immunol..

[2] Jin Y (2004). Mutations of the Wiskott-Aldrich Syndrome Protein (WASP): hotspots, effect on transcription, and translation and phenotype/genotype correlation. Blood.

[3] Thrasher AJ, Burns SO (2010). WASP: a key immunological multitasker. Nat. Rev. Immunol..

[4] Ochs HD, Filipovich AH, Veys P, Cowan MJ, Kapoor N (2009). Wiskott-Aldrich syndrome: diagnosis, clinical and laboratory manifestations, and treatment. Biol. Blood Marrow Transplant..

[5] Sullivan KE, Mullen CA, Blaese RM, Winkelstein JA (1994). A multiinstitutional survey of the Wiskott-Aldrich syndrome. J. Pediatr..

[6] Moratto D (2011). Long-term outcome and lineage-specific chimerism in 194 patients with Wiskott-Aldrich syndrome treated by hematopoietic cell transplantation in the period 1980-2009: an international collaborative study. Blood.

[7] Ozsahin H (2008). Long-term outcome following hematopoietic stem-cell transplantation in Wiskott-Aldrich syndrome: collaborative study of the European Society for Immunodeficiencies and European Group for Blood and Marrow Transplantation. Blood.

[8] Shin CR (2012). Outcomes following hematopoietic cell transplantation for Wiskott-Aldrich syndrome. Bone Marrow Transplant..

[9] Boztug K (2010). Stem-cell gene therapy for the Wiskott-Aldrich syndrome. New Engl. J. Med..

[10] Braun CJ (2014). Gene therapy for Wiskott-Aldrich syndrome–long-term efficacy and genotoxicity. Sci. Transl. Med..

[11] Aiuti A (2013). Lentiviral hematopoietic stem cell gene therapy in patients with Wiskott-Aldrich syndrome. Science.

[12] Ferrua F (2019). Lentiviral haemopoietic stem/progenitor cell gene therapy for treatment of Wiskott-Aldrich syndrome: interim results of a non-randomised, open-label, phase 1/2 clinical study. Lancet Haematol..

[13] Hacein-Bey Abina S (2015). Outcomes following gene therapy in patients with severe Wiskott-Aldrich syndrome. JAMA.

[14] De Ravin, S. S. et al. CRISPR-Cas9 gene repair of hematopoietic stem cells from patients with X-linked chronic granulomatous disease. *Sci. Transl. Med.*10.1126/scitranslmed.aah3480 (2017). 10.1126/scitranslmed.aah3480 28077679

[15] Kuo CY (2018). Site-specific gene editing of human hematopoietic stem cells for X-linked hyper-IgM syndrome. Cell Rep..

[16] Pavel-Dinu M (2019). Gene correction for SCID-X1 in long-term hematopoietic stem cells. Nat. Commun..

[17] Schiroli, G. et al. Preclinical modeling highlights the therapeutic potential of hematopoietic stem cell gene editing for correction of SCID-X1. *Sci. Transl. Med.*10.1126/scitranslmed.aan0820 (2017). 10.1126/scitranslmed.aan0820 29021165

[18] Hendel A (2015). Chemically modified guide RNAs enhance CRISPR-Cas genome editing in human primary cells. Nat. Biotechnol..

[19] Vakulskas CA (2018). A high-fidelity Cas9 mutant delivered as a ribonucleoprotein complex enables efficient gene editing in human hematopoietic stem and progenitor cells. Nat. Med..

[20] Notta F (2011). Isolation of single human hematopoietic stem cells capable of long-term multilineage engraftment. Science.

[21] Burns S, Thrasher AJ, Blundell MP, Machesky L, Jones GE (2001). Configuration of human dendritic cell cytoskeleton by Rho GTPases, the WAS protein, and differentiation. Blood.

[22] Linder S, Nelson D, Weiss M, Aepfelbacher M (1999). Wiskott-Aldrich syndrome protein regulates podosomes in primary human macrophages. Proc. Natl Acad. Sci. USA.

[23] Zicha D (1998). Chemotaxis of macrophages is abolished in the Wiskott-Aldrich syndrome. Br. J. Haematol..

[24] Meyer-Bahlburg A (2008). Wiskott-Aldrich syndrome protein deficiency in B cells results in impaired peripheral homeostasis. Blood.

[25] Westerberg LS (2008). WASP confers selective advantage for specific hematopoietic cell populations and serves a unique role in marginal zone B-cell homeostasis and function. Blood.

[26] Eto K, Kunishima S (2016). Linkage between the mechanisms of thrombocytopenia and thrombopoiesis. Blood.

[27] Haddad E (1999). The thrombocytopenia of Wiskott Aldrich syndrome is not related to a defect in proplatelet formation. Blood.

[28] Kajiwara M (1999). WASP is involved in proliferation and differentiation of human haemopoietic progenitors in vitro. Br. J. Haematol..

[29] Sereni, L. et al. Lentiviral gene therapy corrects platelet phenotype and function in patients with Wiskott-Aldrich syndrome. *J. Allergy Clin. Immunol*. 10.1016/j.jaci.2019.03.012 (2019). 10.1016/j.jaci.2019.03.012 PMC6721834 30926529

[30] Sereni L (2018). Autonomous role of Wiskott-Aldrich syndrome platelet deficiency in inducing autoimmunity and inflammation. J. Allergy Clin. Immunol..

[31] Parkman R (1978). Complete correction of the Wiskott-Aldrich syndrome by allogeneic bone-marrow transplantation. New Engl. J. Med..

[32] Dupre L (2002). Wiskott-Aldrich syndrome protein regulates lipid raft dynamics during immunological synapse formation. Immunity.

[33] Morales-Tirado V (2004). Cutting edge: selective requirement for the Wiskott-Aldrich syndrome protein in cytokine, but not chemokine, secretion by CD4+ T cells. J. Immunol..

[34] Castiello MC (2014). Wiskott-Aldrich Syndrome protein deficiency perturbs the homeostasis of B-cell compartment in humans. J. Autoimmun..

[35] Cradick TJ, Qiu P, Lee CM, Fine EJ, Bao G (2014). COSMID: a web-based tool for identifying and validating CRISPR/Cas off-target sites. Mol. Ther. Nucleic Acids.

[36] Tsai SQ (2015). GUIDE-seq enables genome-wide profiling of off-target cleavage by CRISPR-Cas nucleases. Nat. Biotechnol..

[37] Tsai SQ, Topkar VV, Joung JK, Aryee MJ (2016). Open-source guideseq software for analysis of GUIDE-seq data. Nat. Biotechnol..

[38] Zhu LJ (2017). GUIDEseq: a bioconductor package to analyze GUIDE-Seq datasets for CRISPR-Cas nucleases. BMC Genomics.

[39] Gutierrez-Guerrero A (2018). Comparison of zinc finger nucleases versus CRISPR-specific nucleases for genome editing of the Wiskott-Aldrich syndrome locus. Hum. Gene Ther..

[40] Laskowski TJ (2016). Gene correction of iPSCs from a Wiskott-Aldrich syndrome patient normalizes the lymphoid developmental and functional defects. Stem Cell Rep..

[41] Nesic D, Maquat LE (1994). Upstream introns influence the efficiency of final intron removal and RNA 3′-end formation. Genes Dev..

[42] Genovese P (2014). Targeted genome editing in human repopulating haematopoietic stem cells. Nature.

[43] Astrakhan A (2012). Ubiquitous high-level gene expression in hematopoietic lineages provides effective lentiviral gene therapy of murine Wiskott-Aldrich syndrome. Blood.

[44] Snapper SB (1998). Wiskott-Aldrich syndrome protein-deficient mice reveal a role for WASP in T but not B cell activation. Immunity.

[45] Zhang J (1999). Antigen receptor-induced activation and cytoskeletal rearrangement are impaired in Wiskott-Aldrich syndrome protein-deficient lymphocytes. J. Exp. Med..

[46] Cavazza A, Moiani A, Mavilio F (2013). Mechanisms of retroviral integration and mutagenesis. Hum. Gene Ther..

[47] Notarangelo LD, Miao CH, Ochs HD (2008). Wiskott-Aldrich syndrome. Curr. Opin. Hematol..

[48] Perdomo, J., Yan, F., Leung, H. H. L. & Chong, B. H. Megakaryocyte Differentiation and Platelet Formation from Human Cord Blood-derived CD34+ Cells. *J Vis. Exp*., 10.3791/56420 (2017). 10.3791/56420 PMC5908394 29364213

[49] Pinello L (2016). Analyzing CRISPR genome-editing experiments with CRISPResso. Nat. Biotechnol..

