# Supplementary material for: Targeted gene correction of human hematopoietic stem cells for the treatment of Wiskott - Aldrich Syndrome
Source: Nat Commun. 2020 Aug 12;11:4034. doi: 10.1038/s41467-020-17626-2 (PMC7423939; doi:10.1038/s41467-020-17626-2)
Supplement: Supplementary file 1 — Supplementary Information [file 41467_2020_17626_MOESM1_ESM.pdf]

Targeted gene correction of human hematopoietic stem cells for the treatment of Wiskott-Aldrich Syndrome

Rai et al.

Address for correspondence:
Dr Alessia Cavazza
UCL Great Ormond Street Institute of Child Health
30 Guilford Street
London
WC1N 1EH
email: a.cavazza@ucl.ac.uk

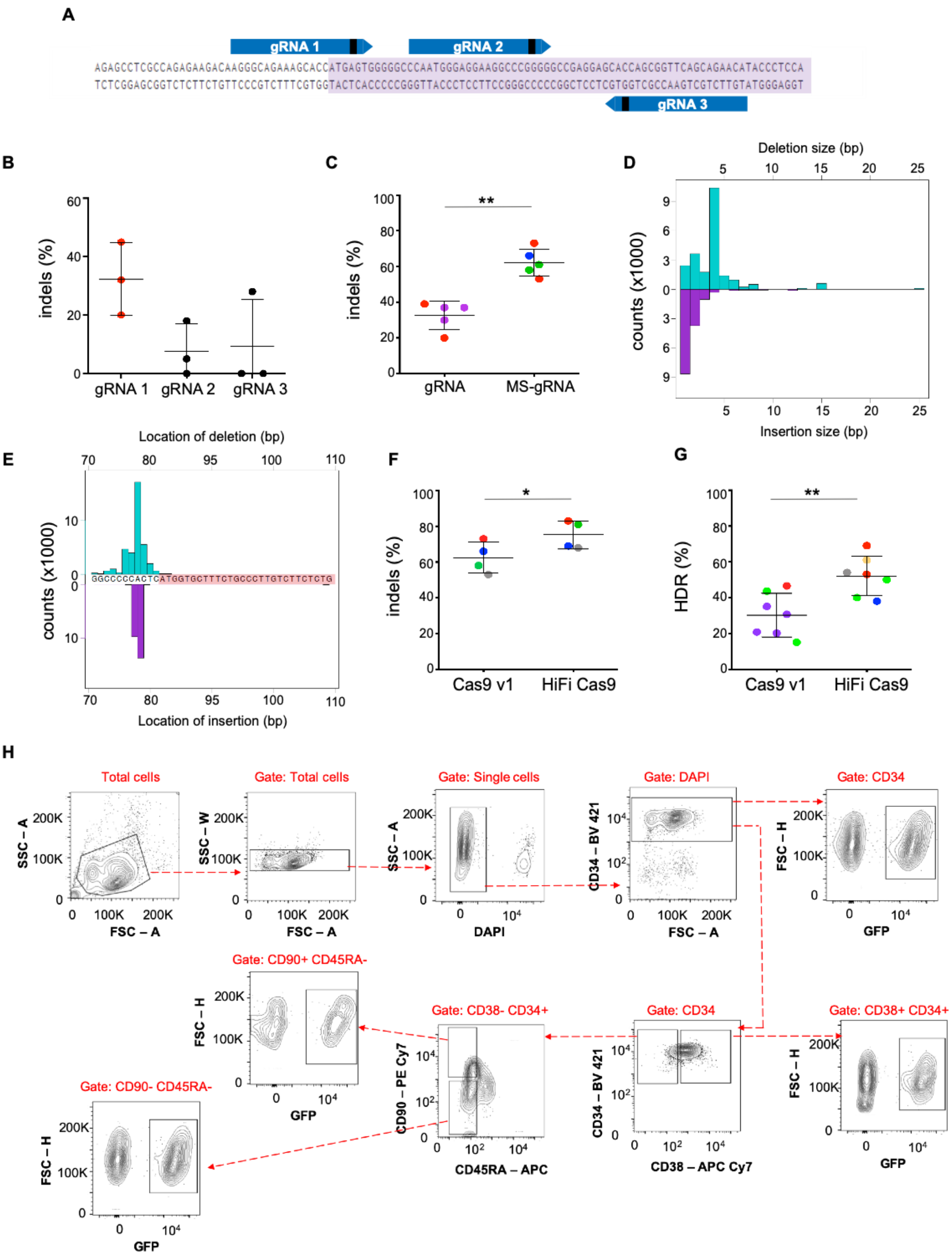
A
gRNA 1
gRNA 2
gRNA 3
B
C
D
E
F
G
H
indels (%)
gRNA 1
gRNA 2
gRNA 3
gRNA
MS-gRNA
Deletion size (bp)
Insertion size (bp)
counts (x1000)
Location of deletion (bp)
Location of insertion (bp)
Cas9 v1
HiFi Cas9
HDR (%)
Total cells
Gate: Total cells
Gate: Single cells
Gate: DAPI
Gate: CD34
Gate: CD90+ CD45RA-
Gate: CD38- CD34+
Gate: CD34
Gate: CD38+ CD34+
Gate: CD90- CD45RA-
SSC – A
SSC – W
FSC – A
FSC – H
DAPI
CD34 – BV 421
GFP
CD90 – PE Cy7
CD45RA – APC
CD38 – APC Cy7

Supplementary Figure 1. Development of a stem cell gene editing platform targeting WAS. A) Schematics of the binding sites of the three gRNAs targeting the *WAS* genomic locus. The purple box indicates the WASp protein coding sequence, starting from the translation start site (ATG). **B)** K562 cells were electroporated with each of the candidate gRNA complexed with the Cas9 protein and INDELs were analysed by TIDE software (n=3 experiments). **C)** HSPCs were electroporated with either unmodified or chemically modified (MS) gRNA complexed with Cas9 and INDELs were analysed using TIDE software (n=5 experiments, coloured dots represent different PB donors; **p=0.004, by two-tailed paired Student's t-test). **D**) Quantification of the size and frequency of deletions and insertions introduced by the NHEJ-mediated repair of the cut site at the *WAS* locus by targeted high-throughput sequencing. Number or reads per type of modification detected are indicated on the y axis. **E**) Localization of deletions and insertions introduced by the NHEJ-mediated repair with respect to the cut site at the *WAS* locus. The sequence highlighted in pink represent WAS coding sequence. **F)** HSPCs were electroporated with the chemically modified (MS) gRNA complexed with either Cas9 v1 or HiFi Cas9; and INDELs were analysed using TIDE software (n=4 experiments, coloured dots represent different PB donors; *p=0.05, by two-tailed paired Student's t-test). **G)** Rates of targeted integration (HDR) achieved in HSPCs after electroporation with the MS-gRNA and either Cas9 v1 or HiFi Cas9, as assessed by flow cytometry (n=7 experiments, coloured dots represent different PB donors; **p=0.005, by two-tailed paired Student's t-test). **H)** Gating strategy for the sorting of HSCs (CD34+ CD38-CD90+ CD45RA-), MPPs (CD34+ CD38-CD90- CD45RA-) and CD38+(CD34+ CD38+) cell populations from bulk CD34+ HSPCs cells (Figure 1I-L).

Data are presented as mean ± SD. Source data are provided as a Source Data file.

A

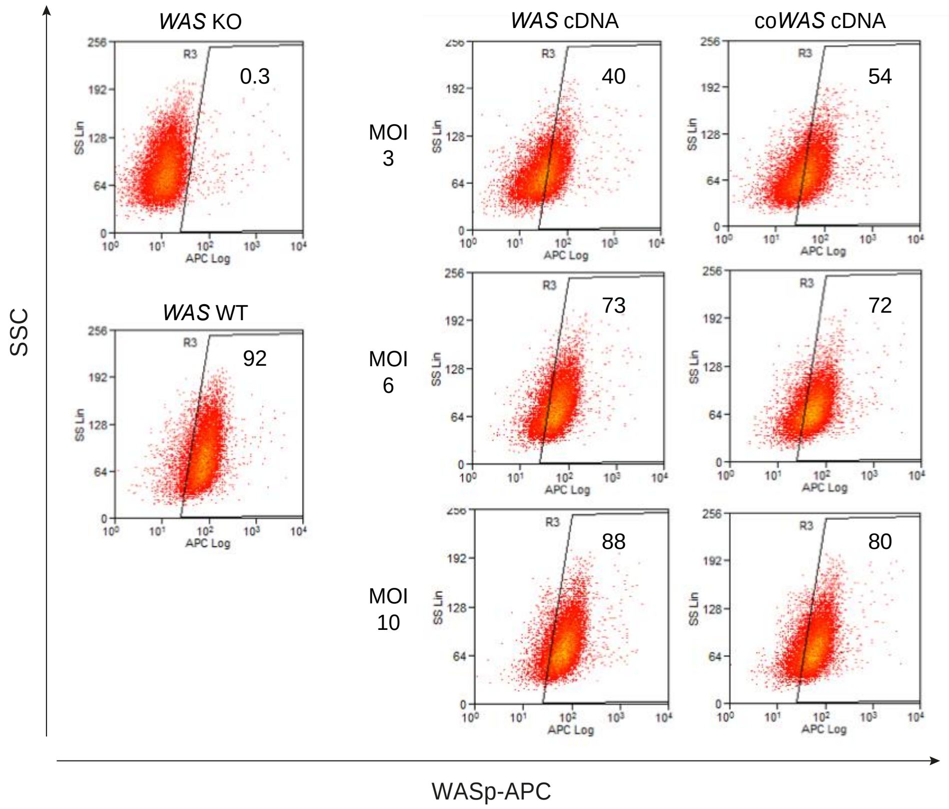

B

Patient	Exon	Type of mutation	Nucleotide mutation	Amino acid effect	HSPC source
**WAS01**	12	Deletion	1483 del G	Asp 495 Met/ Frameshift 164 Stop	PB
**WAS02**	1	Nonsense	C 97 T	Gln 33 Stop	PB
**WAS03**	4	Missense	G 391 A	Glu 131 Lys	PB
	9	Nonsense	C 913 T	Gln 305 Stop	PB
**WAS04**	1	Nonsense	C 100 T	Arg 34 Stop	PB
**WAS05**	3	Missense	T 302 C	Leu 101 Pro	BM

Supplementary Figure 2. Application of the CRISPR/Cas9 platform to WAS patient-derived HSPCs. A) Analysis of WASp expression mediated by either a *WAS* wild-type cDNA (*WAS* cDNA) or the codon optimized *WAS* cDNA used in our gene editing approach. The cDNAs were cloned in a lentiviral vector and used to transduce a *WAS* knock-out THP-1 cell line at different MOIs. WASp expression was detected by flow cytometry. Percentage of

WASp+ cells is indicated in each gate. Untransduced WAS knock-out THP-1 cells (WAS KO) or wild-type THP-1 cells (WAS WT) are used as a negative and positive control for antibody staining, respectively. **B)** Description of *WAS* mutations detected in WAS patients' cells used in this study.

A

Replicate		M1	M2	M3	M4	M5	M6	M7	M8
Patient		WAS02	WAS01	WAS02	WAS02	WAS01	WAS02	WAS03	WAS04
Sample	WT HSPC	32.9	29.7	34.7	46.2	36.8	25	37	37
	WAS HSPC	46.8	44.4	33.3	51.9	34.3	25	38.9	51.9
	WAS W_pA	42.7	53.1	50	66.7	57.1	50	33.3	41.7
	WAS W_UTR		48.2	52.2		31.8	-	41.4	58.9
	WAS WW1.6	22.6	13.8	40	40	33.3	25	44.4	62.5

B

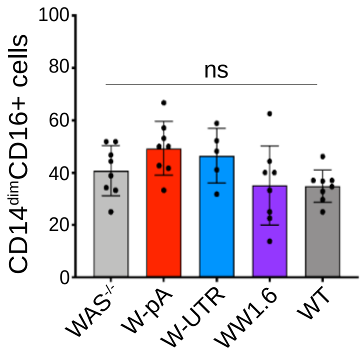

C

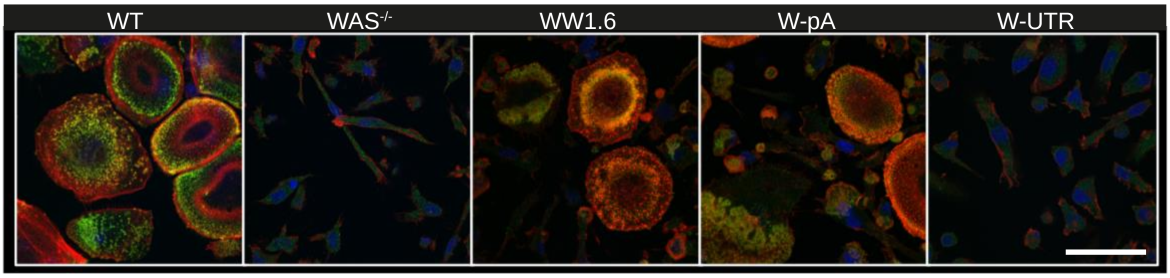

Supplementary Figure 3. Correction of the functional defects in WAS macrophages. A) Percentage of CD14+ monocytes obtained from the in vitro differentiation of WT and WAS HSPCs for each experimental condition (NS, not significant; one-way ANOVA with Bonferroni's multiple comparison test). **B)** Average percentage of CD14dimCD16+ macrophages obtained from the *in vitro* differentiation of WT and WAS HSPCs for each experimental condition (n=8 independent experiments for all groups except for W-UTR (n=5) from 4 different donors; data are presented as mean ± SD; NS, not significant; one-way ANOVA with Bonferroni's multiple comparison test). **C)** Representative composite images of

podosomes by confocal microscopy at 63x magnification. For each condition, fibronectin-adhering macrophages were stained with DAPI (blue) and with antibodies against Vinculin (green) and F-actin (red). Five independent experiments were performed. Scale bar 10 μm, for all panels. Source data are provided as a Source Data file.

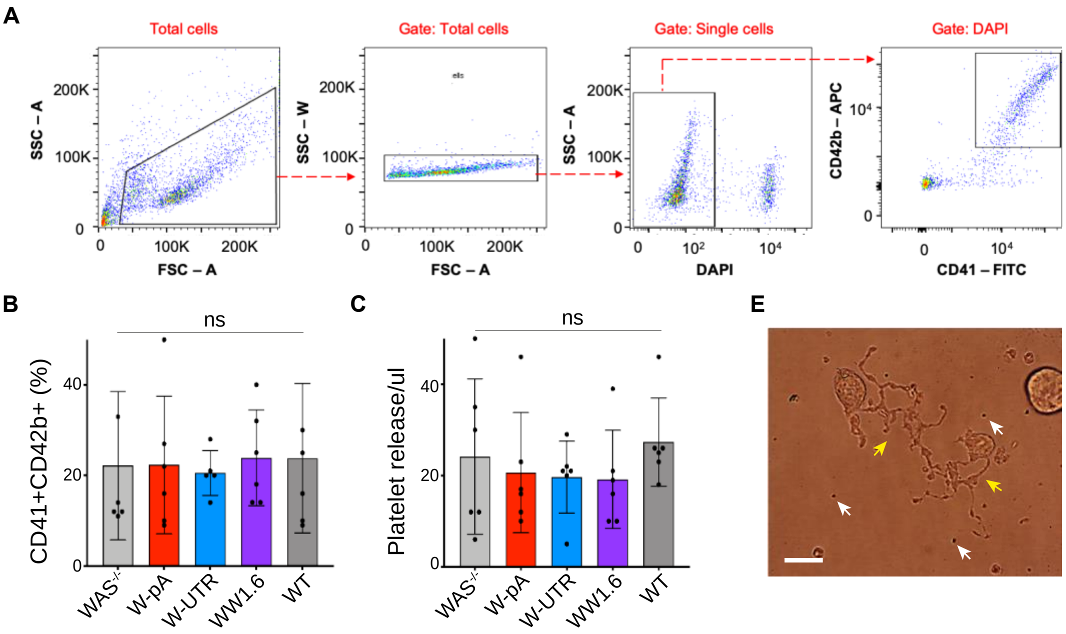

D

Replicate		P1	P2	P3	P4	P5	P6
Patient		WAS01	WAS02	WAS01	WAS02	WAS03	WAS04
Sample	WT HSPC	56	25	43	18	26	26
	WAS HSPC	80	6	12	12	35	30
	WAS W_pA	46	12	16	17	10	23
	WAS W_UTR	5	21	20	21	29	22
	WAS WW1.6	39	10	10	16	19	21

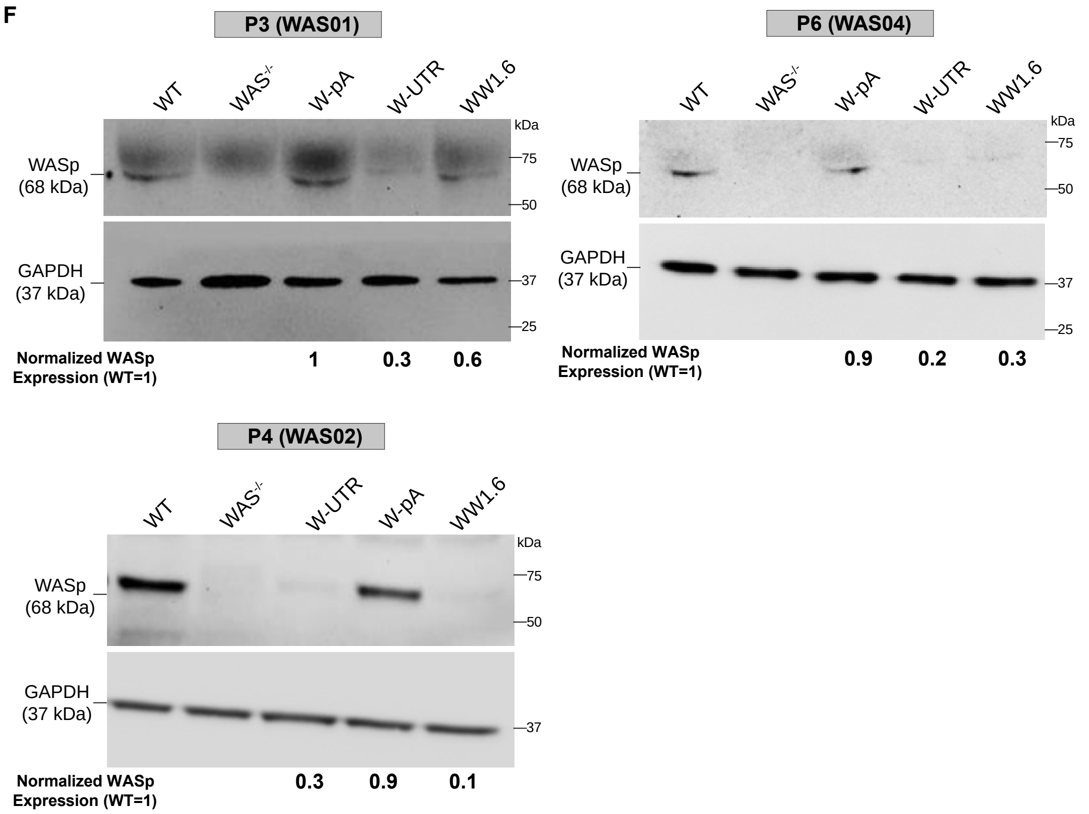

Supplementary Figure 4. Correction of the functional defects in WAS platelets. A) Gating strategy for the analysis of megakaryocytic progenitors *in vitro* differentiated from HSPCs, for the data presented in Figure 3G,H and Supplementary Figure 4B,C. **B)** Percentage of megakaryocytic progenitors (CD41+CD42b+) obtained from the in vitro differentiation of WT and WAS HSPCs for each experimental condition (n=6 independent experiments for all groups except for W-UTR (n=5) from 4 different donors. Data are presented as mean ± SD; NS, not significant; one-way ANOVA with Bonferroni's multiple comparison test). **C and D)** Platelet release per microliter obtained from the in vitro differentiation of WT and WAS HSPCs for each experimental condition (n=6 independent experiments for all groups except for W-UTR (n=5) from 4 different donors. Data are presented as mean ± SD; NS, not significant; one-way ANOVA with Bonferroni's multiple comparison test). **E)** Representative picture of proplatelet-bearing megakaryocytes (yellow arrows) and platelets (white arrows) obtained by in vitro differentiation of PB CD34+HSPCs. Six independent experiments were performed. Scale bar 5 μm. **F)** WASp expression detected by immunoblotting in HSPC-derived platelets for each experimental condition; GAPDH was used as a loading control for protein normalization. WASp expression was analysed by densitometry and normalized to GAPDH loading control. Each blot represents a biologically independent experiment (n=3 in total).

Source data are provided as a Source Data file.

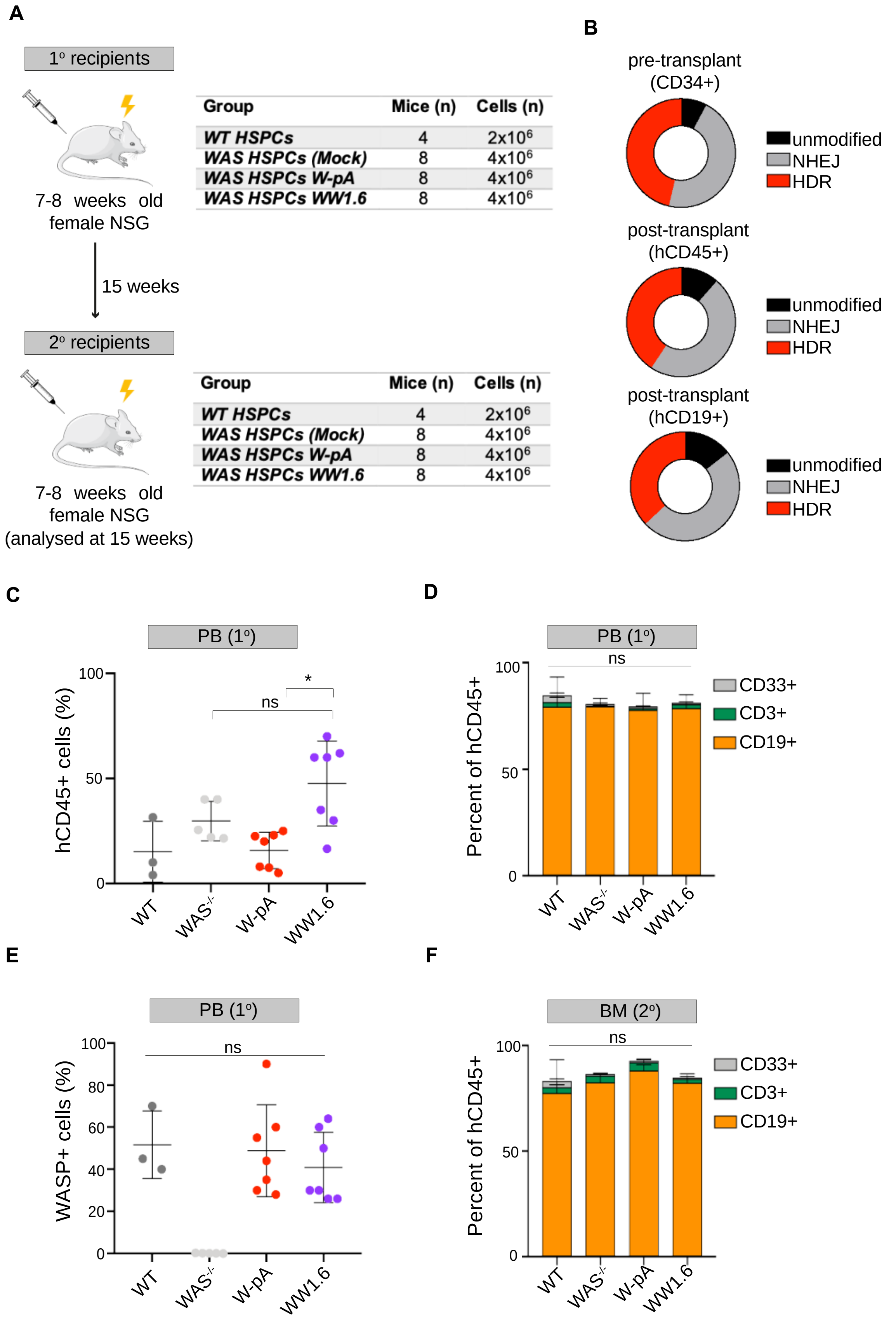
A
1º recipients
7-8 weeks old female NSG
Group | Mice (n) | Cells (n)
WT HSPCs | 4 | 2x10^6
WAS HSPCs (Mock) | 8 | 4x10^6
WAS HSPCs W-pA | 8 | 4x10^6
WAS HSPCs WW1.6 | 8 | 4x10^6
15 weeks
2º recipients
7-8 weeks old female NSG (analysed at 15 weeks)
Group | Mice (n) | Cells (n)
WT HSPCs | 4 | 2x10^6
WAS HSPCs (Mock) | 8 | 4x10^6
WAS HSPCs W-pA | 8 | 4x10^6
WAS HSPCs WW1.6 | 8 | 4x10^6
B
pre-transplant (CD34+)
post-transplant (hCD45+)
post-transplant (hCD19+)
unmodified
NHEJ
HDR
C
PB (1º)
hCD45+ cells (%)
ns
*
WT
WAS-/-
W-pA
WW1.6
D
PB (1º)
Percent of hCD45+
ns
CD33+
CD3+
CD19+
E
PB (1º)
WASP+ cells (%)
ns
F
BM (2º)
Percent of hCD45+
ns
CD33+
CD3+
CD19+

Supplementary Figure 5. *In vivo* hematopoietic reconstitution by gene targeted WAS HSPCs. A) Overview of the *in vivo* transplantation experiments; the number of mice per experimental group and total number of WT or WAS HSPCs transplanted are indicated. This figure was created using Servier Medical Art templates, which are licensed under a Creative Commons Atrtribution 3.0 Unported License; https://smart.servier.com. **B)** Characterization of allelic modification (HDR-, NHEJ-corrected or non-modified alleles) in HSPCs cells before transplant and in engrafted hCD45+ cells or CD19+ B-cells isolated from mice after transplant. The frequency of each allelic modification was obtained by ddPCR and targeted deep-sequencing. **C)** Engraftment of human cells (hCD45+) in the PB of NSG mice 14 weeks after primary transplant (n=7 mice for all groups except for WT (n=3) and WAS-/- (n=5); *p=0.03; NS, not significant; two-way ANOVA with Bonferroni's multiple comparison test). **D)** Lineage distribution of hCD45+ human cells engrafted in the PB of NSG mice 14 weeks after transplant; T-cells: CD3+; B-cells: CD19+; myeloid cells: CD33+ (n=7 mice for WW1.6, n=6 for W-pA, n=5 for WAS-/-, n=3 for WT; NS, not significant; two-way ANOVA with Bonferroni's multiple comparison test). **E)** Percentage of WASp expressing cells detected by flow cytometry in hCD45+ cells harvested from the PB of primary transplanted animals (n=7 mice for WW1.6 and W-pA, n=5 for WAS-/-, n=3 for WT; NS, not significant; two-way ANOVA with Bonferroni's multiple comparison test). **F)** Lineage distribution of hCD45+ human cells engrafted in the BM of NSG mice 12 weeks after secondary transplant; T-cells: CD3+; B-cells: CD19+; myeloid cells: CD33+ (n=7 mice for all groups except for WT (n=4) and WAS-/- (n=5); NS, not significant; two-way ANOVA with Bonferroni's multiple comparison test). Data are presented as mean ± SD. Source data are provided as a Source Data file.

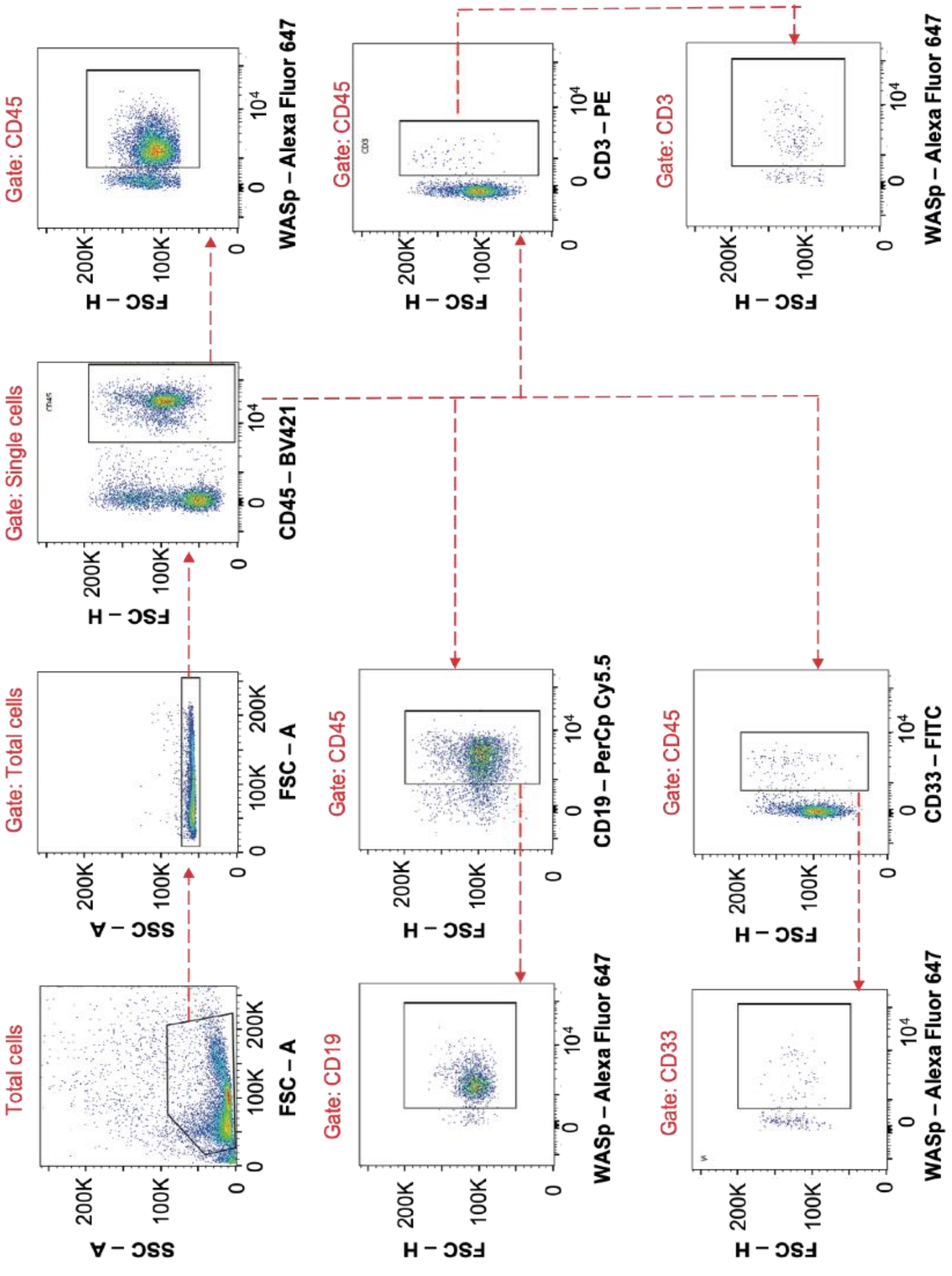

Supplementary Figure 6. Gating strategy for multilineage analysis. The following gating strategy was used to analyse multilineage development *in vivo* (data presented in Figure 5A-D and Supplementary Figure 5C-F).

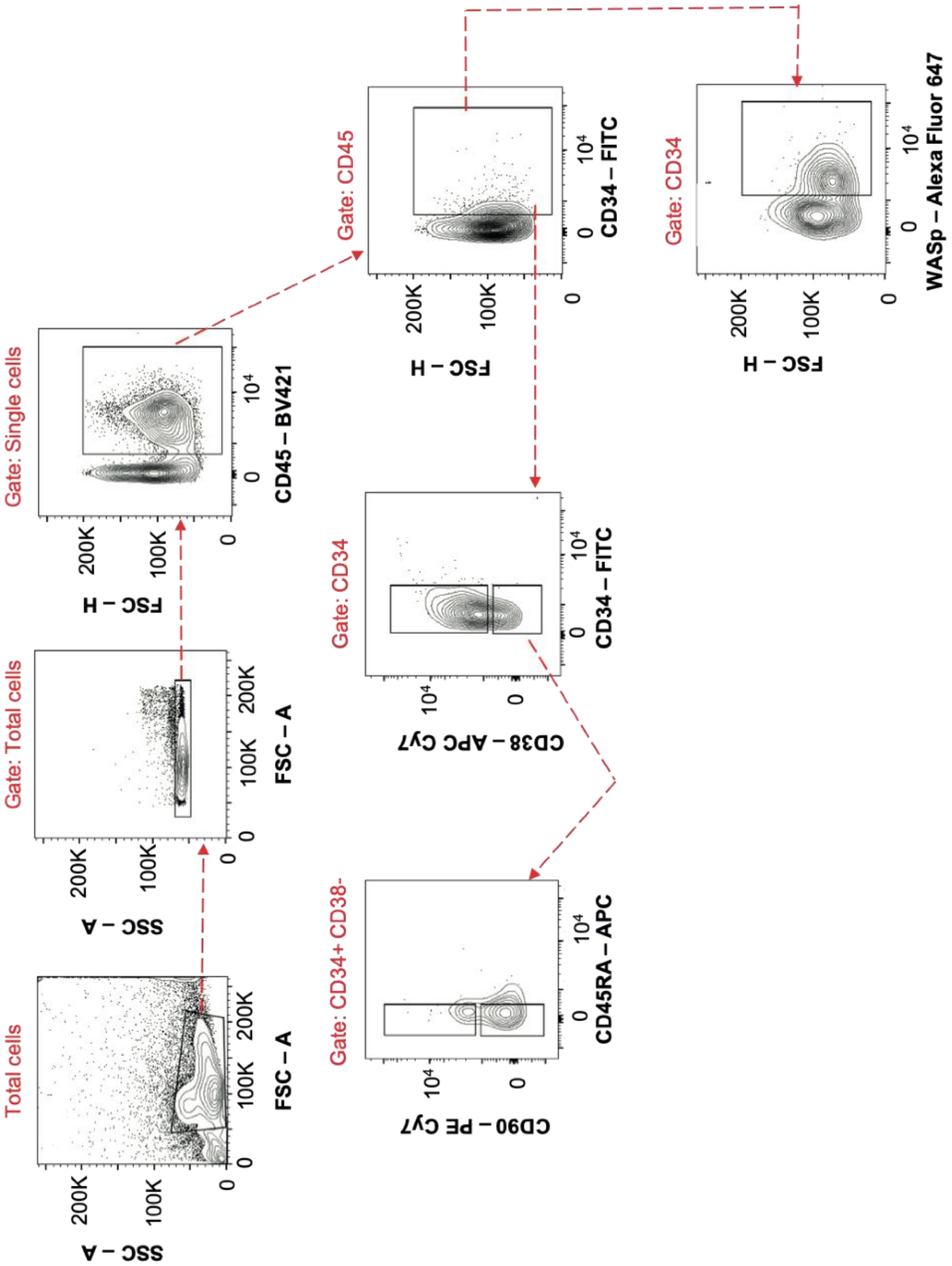

Supplementary Figure 7. Gating strategy for the analysis of stem cell subpopulations in the bone marrow of transplanted mice. The following gating strategy was used to analyze the composition of different stem cell populations (HSCs, MPPs and CD38+ cells) in the bone marrow of transplanted mice (data presented in Figure 5E, F).

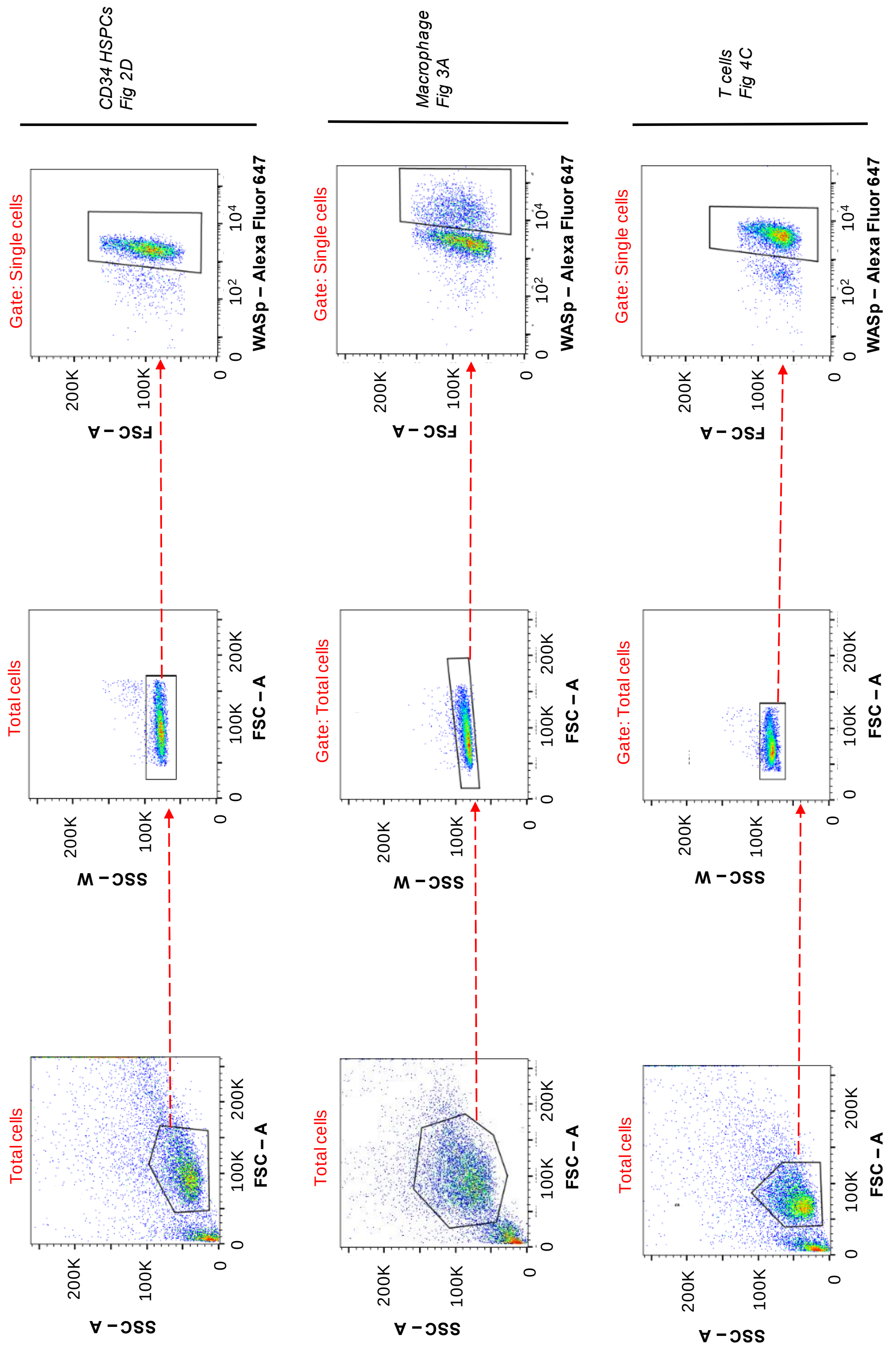

Supplementary Figure 8. Gating strategy for the analysis of WASp expression in different cell types. The following gating strategy was used to analyze the expression of WASp in HSCPs, Macrophages and T-cells in in vitro experiments (data presented in Figure 2D and Supplementary Figure 2, Figure 3A, 4C, respectively).

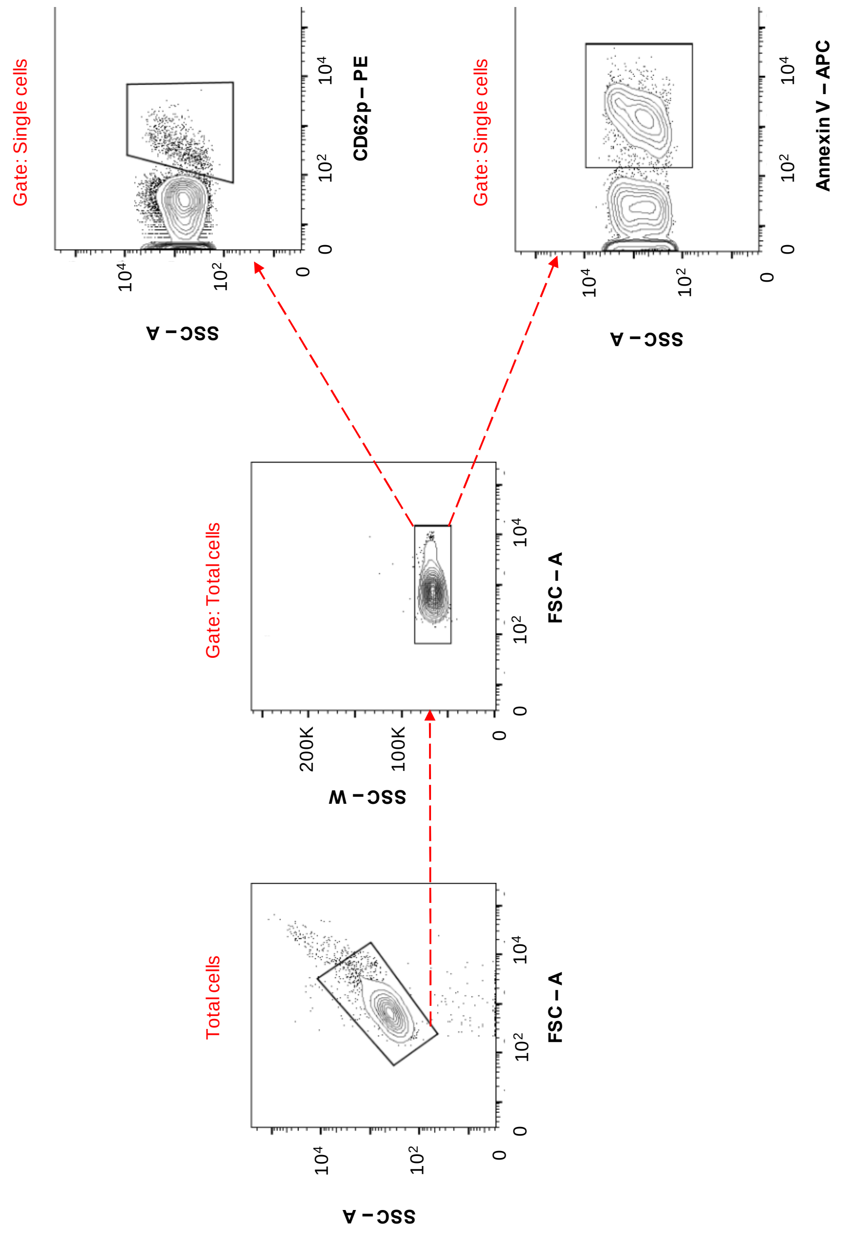

Supplementary Figure 9. Gating strategy for the analysis of platelets. The following gating strategy was used to analyze the size, granularity, activation and apoptotic profiles of HSPC-derived platelets (data presented in Figure 3F-I).

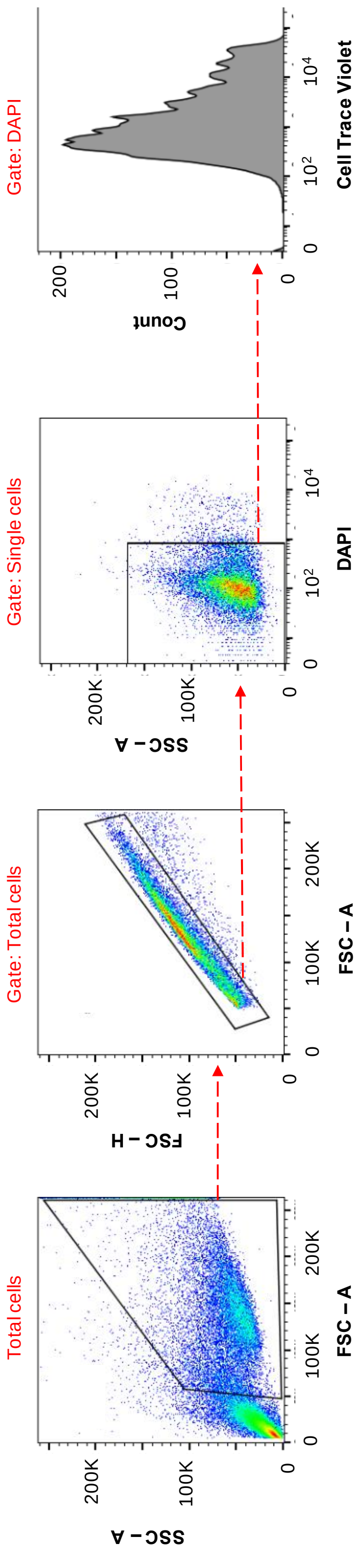

Supplementary Figure 10. Gating strategy for the analysis of T-cell proliferation. The following gating strategy was used to analyze the proliferation ability of WAS T-cell before and after gene editing (data presented in Figure 4F).

A

					! "#" $%&			! "#" $ &		
( ) $* +, &	- . $&	/ 0 12) , 3. &	4 +#+&	5+) , 6$+&	7 8 9&	/ : - ; &	<=, +1, &	7 8 9&	/ : - ; &	<=, +1, &
: (%&	! "	#"	$ %&' #( ) "	*+,-. +"	/ 0 1"	/ 0 1"	2 30 4! "	/ 0 1"	/ 0 #"	2) ' 0 "
: (' &	) 3"	#"	"	*+,5-65+7 8"	/ 0) "	/ 0 ' "	2) ! 0 93"	/ 0) "	/ 0 ' "	2#: 0 3' "
: (>&	3"	#"	"	*+,5-65+7 8"	/ 0) "	/ 0) "	2) ! 0 4"	/ 0) "	/ 0 ' "	2#/ 0 / "
: (?&	9"	#"	"	*+,5-65+7 8"	/ 0 ' "	/ 0) "	2) 10) "	/ 0 #"	/ 0 ' "	2 #0 3/ "
: (@&	) "	#"	"	*+,5-65+7 8"	/ 0 ! "	/ 0 1"	2 1 0#' "	/ 0 3"	/ 0 3"	2 9 0 9#"
: (A&	' ' "	; 5<! "	=> ? @A' "	*+,-. +"	/ 0 3"	/ 0 1"	2#0#3"	/ 0 : "	/ 0 9"	2 3 0) "
: (B&	1"	#"	$ %> *B "	*+,-. +"	/ 0 #"	/ 0 #"	2 1 0 9' "	/ 0 ' "	/ 0 #"	2) ! 0 ' "
: (C&	) "	#"	"	*+,5-65+7 8"	/ 0 ' "	/ 0) "	2) ' 0 1#"	/ 0 ' "	/ 0 ' "	2 ' 0 4#"
: (D&	' "	#"	=C(D) 3"	*+,-. +"	/ 0) "	/ 0 ' "	2) ! 0 14"	/ 0 ' "	B("	B("
: (%E&	) / "	#"	=EAE"	*+,-. +"	/ 0) "	/ 0) "	2) #0##"	/ 0 / "	/ 0) "	2#4 0 91"
: (%%&	' / "	#"	"	*+,5-65+7 8"	/ 0 ' "	/ 0) "	2 9 0) "	/ 0 ' "	B("	B("
: (% &	' / "	#"	"	*+,5-65+7 8"	/ 0 1"	) 0 9"	2) 1 0 3#"	/ 0 #"	/ 0 9"	2) 0 1"
: (%>&	)) "	#"	=F (B G' "	*+,-. +"	/ 0 ! "	/ 0 / "	2/ 0) "	/ 0#! "	/ 0#) "	2#0 1: "
: (%?&	)) "	#"	"	*+,5-65+7 8"	/ 0 #"	/ 0 4"	2 1 0 3' "	/ 0 4"	/ 0 : "	2) 3 0#! "
: (%@&	#"	) "	"	*+,5-65+7 8"	/ 0 ' "	/ 0 ' "	2 ' 0 14"	/ 0 ' "	/ 0 ' "	2#/ 0 9"

!

B

Off-target	Chromosome	Position	Reads (n)	Strand	Feature	Gene	Off-Target Sequence	Mismatches
**OT1-G**	chrX	124413678	6	+	intron 17	TENM1	GCATAAAGCACAAGG AGAGGTGA	5
**OT2-G**	chr2	197121391	5	+	exon 9	ANKRD44	ACACAAAGCACCATG AGTGGAGG	2
**OT3-G**	chr3	2220064	5	-	intron 1	CNTN4	GGAGAAAACACTCTG AGTGATGG	5
**OT4-G**	chr6	159803437	2	-	intron 4	PNLDC1	GCAGAGGGCAGAGT GAGTGGAGG	5
**OT5-G**	chr1	50322324	1	+	intergenic		GGAAACAGCACCCT GAGAGGAGG	5
**OT6-G**	chr2	112824054	1	-	intron 1	AC079753. 2 (ncRNA)	GCAGCATGAACAATG GGTGGAGG	5

Supplementary Figure 11. Genotoxicity analysis in edited HSPCs. A) Description of off-target sites detected by COSMID and indels frequencies quantified by targeted high-throughput sequencing in mock and edited HSPCs derived from 2 different healthy donors. **B)** Description of off-target sites detected by GUIDE-seq.

Host	Antibody	Fluorochrome	Clone	Company	Catalog #
**Mouse**	anti-human CD45	APC	HI30	BD Bioscience	555485
**Mouse**	anti-human CD45	BV 421	HI30	Biolegend	304032
**Mouse**	anti-human CD3	PE	OKT3	Biolegend	317308
**Mouse**	anti-human CD33	FITC	P67.6	BD Bioscience	345798
**Mouse**	anti-human CD19	PerCp Cy5.5	HIB19	Biolegend	302230
**Mouse**	anti-human WASP	-	5A5	BD Bioscience	557773
**Goat**	anti-mouse IgG	Alexa Fluor 647	Poly4053	Biolegend	405322
**Rabbit**	anti-human WASp	-	EP2541Y	Abcam	ab75830
**Donkey**	anti-rabbit WASp	Alexa Fluor 647	Poly4064	Biolegend	406414
**Mouse**	anti-human CD34	BV 421	561	Biolegend	343610
**Mouse**	anti-human CD34	FITC	561	Biolegend	343604
**Mouse**	anti-human CD38	APC Cy7	HIT2	Biolegend	303534
**Mouse**	anti-human CD90	PE Cy7	5E10	Biolegend	328124
**Mouse**	anti-human CD45RA	APC	HI100	Biolegend	304112
**Mouse**	anti-human CD45RA	PerCp Cy5.5	HI100	Biolegend	304122
**Mouse**	anti-human CD41a	FITC	HIP8	Biolegend	303704
**Mouse**	anti-human CD42b	APC	HIP8	BD Bioscience	551061
**Mouse**	anti-human CD61	APC	VI-PL2	Biolegend	336412
**Mouse**	anti-human CD62p	PE	VI-P44	Biolegend	304906
**Mouse**	anti-human CD3	-	OKT3	Biolegend	317302
**Mouse**	anti-human Vinculin	-	V4505	Sigma-Aldrich	V4505
**Goat**	anti-mouse IgG	Alexa Fluor 488	H+L	ThermoFisher Scientific	A28175
	Annexin V	APC		BD Bioscience	550474
**Mouse**	anti-human GAPDH		0411	Santa Cruz	sc-47724
**Sheep**	anti-mouse IgG	HRP		GE Healthcare	NXA931
**Mouse**	anti-human CD14	PerCp Cy5.5	G1D3	ThermoFisher Scientific	45-0149-42
**Mouse**	anti-human CD16	APC	CB16	ThermoFisher Scientific	17-0168-41
	Phalloidin	Alexa Fluor 635		ThermoFisher Scientific	A34054

Supplementary Table 1. List of antibodies used in this study.

Assay	*Primer name*	*Sequence (5' - 3')*
ddPCR	WAS-pA_for	TTGGGAAGAGAATAGCAGGC
	WAS-pA_rev	GGCAGCAGACTAATACTGGG
	WAS-UTR_for	AGAATTGTCTTTCTGTCTCTCT
	WAS-UTR_rev	GGCAGCAGACTAATACTGGG
	Albumin-reference_for	GCTGCTATCTCTTGTGGGCTGT
	Albumin-reference_rev	ACTCATGGGAGCTGCTGGTTC
	WW1.6-PSI_for	CAGGACTCGGCTTGCTGAAG
	WW1.6-PSI_rev	TCCCCCGCTTAATACTGACG
	FAM probe_WAS	TGGCGCTGCCCCCTGGAGCT
	HEX probe_Albumin	CCTGTCATGCCCACACAAATCTCTCC
	FAM probe_PSI	CGCACGGCAAGAGGCGAGG
Off-targets detection	OT1_for	TGGAAGGGCTTTGGGAGATGAAGG
	OT1_rev	CAGAGCAGAGCGAGACCATG
	OT2_for	GCCACACAGGCCATGATAACAG
	OT2_rev	TAACTTCTGCCACGGCAAGCAGC
	OT3_for	CTGCTAATGGCGCAGGTATAGCAA
	OT3_rev	ACACCCATGATGTTGGGAACCAG
	OT4_for	CCTGTAGGGAGAGGGACTGA
	OT4_rev	CCCACCTTTTCTCCTGCATCTG
	OT5_for	GACCCACTTACTGGGCTTTTAAGG
	OT5_rev	GAGACTATCTGACCAGCAAGCC
	OT6_for	GCTTTACTTTCAGGTACCTGTGGG
	OT6_rev	CTCTGTGGAAGGACAAGGACC
	OT7_for	CTTATGAAGGTGCTTTTTTGTGTGGATGCTG
	OT7_rev	CCTGGAGTACTAATTAAACTCATCTCTGAGTTC
	OT8_for	TGCTCCCTATTCTGTGAGTGCTTG
	OT8_rev	GTTTCTACCAGGACTCTCCAAGG
	OT9_for	CCGCTCTCCTTGGAAAAACTTGTC
	OT9_rev	CTCATGGGCATCCTAACACTCC
	OT10_for	CCTCCCACCCAGCCAAGTAA
	OT10_rev	GCATCCACACAGCACCACTTC
	OT11_for	CAGAGGGTGTTTCACAAAACAGGC
	OT11_rev	CTCTCTTGGAGCCTGGGCTA
	OT12_for	AGGGGTCAGGTGAGAGAGGT
	OT12_rev	TCAAGGGCAGCCCATCCTTC
	OT13_for	AAGCCACAGAGAGAGAGGGAGAAAG
	OT13_rev	GCACAGCATGGCTTCTGGAG
	OT14_for	GTGCCCTTTCCAAGAAAGAAGACC
	OT14_rev	GGGCTGAATCCTGTAGCCAGT
	OT15_for	CCTGCTCCACAGAGACTGTGA
	OT15_rev	CTCCGTCAGTGATTCTTTCAGCTC
On-target INDEL detection	WAS_genomic_for	TCAGGCTACCTAGGTGCTTTAG
	WAS_genomic_rev	CTGGGAAGGGTGGATTATGAC

Supplementary Table 2. List of primers and probes used in this study.

Biological replicate	Sample type	Cell Nº before electroporation	Cell Nº at day 2 post-editing			
			HSC sorted	MPP sorted	CD38 sorted	CD34 Unsorted
#1	Mock edited	200,000	700,000	580,000	480,000	360,000
	RNP+AAV	200,000	600,000	100,000	360,000	310,000
#2	Mock edited	200,000	700,000	950,000	790,000	530,000
	RNP+AAV	200,000	500,000	120,000	300,000	650,000
#3	Mock edited	200,000	430,000	320,000	780,000	640,000
	RNP+AAV	200,000	1,000,000	460,000	150,000	300,000

Supplementary Table 3. Cell yields before and after gene editing. HSCs, MPPs and CD38+ cells were sorted from bulk CD34+ populations (n=3) immediately after thawing. Cells were plated and 48 hours later 200,000 cells per each condition were electroporated and transduced with the AAV donor vector. Two days post editing, live cells were counted on the flow cytometer using counting beads.
