# Supplementary material for: Targeted gene correction of human hematopoietic stem cells for the treatment of Wiskott - Aldrich Syndrome
Source: Nat Commun. 2020 Aug 12;11:4034. doi: 10.1038/s41467-020-17626-2 (PMC7423939; doi:10.1038/s41467-020-17626-2)
Supplement: Supplementary file 2 — Reporting Summary [file 41467_2020_17626_MOESM2_ESM.pdf]

nature research

Corresponding author(s): Alessia Cavazza

Last updated by author(s): Jun 3, 2020

Reporting Summary

Nature Research wishes to improve the reproducibility of the work that we publish. This form provides structure for consistency and transparency in reporting. For further information on Nature Research policies, see Authors & Referees and the Editorial Policy Checklist .

Statistics

For all statistical analyses, confirm that the following items are present in the figure legend, table legend, main text, or Methods section.

n/a	Confirmed	
☐	☒	The exact sample size (*n*) for each experimental group/condition, given as a discrete number and unit of measurement
☐	☒	A statement on whether measurements were taken from distinct samples or whether the same sample was measured repeatedly
☐	☒	The statistical test(s) used AND whether they are one- or two-sided<br>*Only common tests should be described solely by name; describe more complex techniques in the Methods section.*
☒	☐	A description of all covariates tested
☐	☒	A description of any assumptions or corrections, such as tests of normality and adjustment for multiple comparisons
☐	☒	A full description of the statistical parameters including central tendency (e.g. means) or other basic estimates (e.g. regression coefficient) AND variation (e.g. standard deviation) or associated estimates of uncertainty (e.g. confidence intervals)
☐	☒	For null hypothesis testing, the test statistic (e.g. *F*, *t*, *r*) with confidence intervals, effect sizes, degrees of freedom and *P* value noted<br>*Give P values as exact values whenever suitable.*
☒	☐	For Bayesian analysis, information on the choice of priors and Markov chain Monte Carlo settings
☒	☐	For hierarchical and complex designs, identification of the appropriate level for tests and full reporting of outcomes
☒	☐	Estimates of effect sizes (e.g. Cohen's *d*, Pearson's *r*), indicating how they were calculated

Our web collection on statistics for biologists contains articles on many of the points above.

Software and code

Policy information about availability of computer code

Data collection

1. Benchling was used for planning, visualizing, and documenting cloning projects
2. QuantaSoft (Bio-Rad) was used to analyze ddPCR data
3. BD FACSDIVA (Becton Dickinson) software was included in the BD FACSaria II and BD LSR II analyzer and was used to collect flow cytometry data
4.GeneGnome chemiluminiscence imaging system (SynGene) was used for signal detection in immunoblotting experiments
5. Confocal images were acquired using Zen software (Zeiss).

Data analysis

1. FlowJo (v10) was used for analysis of flow cytometry data
2. Images were quantified using imageJ (v1.52r)
3. COSMID (https://crispr.bme.gatech.edu/) is publicly available web-based tool and was used to predict off-target sites
4. TIDE (v2.0.1) was used for quantifying INDEL rates
5. CRISPResso2 (http://crispresso.pinellolab.partners.org/) was used to quantify INDELs and targeted integration from MiSeq reads
6. GraphPad Prism (v7) was used for graphing and statistical analysis
7. Guide-seq Bioconductor package (v1.18.0) and guide-seq Python package (v3) were used for Guide-seq data analysis

For manuscripts utilizing custom algorithms or software that are central to the research but not yet described in published literature, software must be made available to editors/reviewers. We strongly encourage code deposition in a community repository (e.g. GitHub). See the Nature Research guidelines for submitting code & software for further information.

Data

Policy information about availability of data

All manuscripts must include a data availability statement. This statement should provide the following information, where applicable:

- Accession codes, unique identifiers, or web links for publicly available datasets
- A list of figures that have associated raw data
- A description of any restrictions on data availability

All data that supports conclusion stated are included in the manuscript.

Field-specific reporting

Please select the one below that is the best fit for your research. If you are not sure, read the appropriate sections before making your selection.

☒ Life sciences ☐ Behavioural & social sciences ☐ Ecological, evolutionary & environmental sciences

For a reference copy of the document with all sections, see nature.com/documents/nr-reporting-summary-flat.pdf

Life sciences study design

All studies must disclose on these points even when the disclosure is negative.

Sample size	No sample size calculations were performed. The number of animals used per transplant were determined by the number of cells successfully modified from a single human source divided by the intended dose. The minimum number of mice per condition was determined through a pilot study which provided us with information about the standard deviation and the magnitude of the effects. In addition, the sample size used in this experiment is consistent with previous reports (Pavel-Dinu et al., 2019, Nature Comm; Gomez-Ospina et al., 2019, Nature Comm). In fact, the resulting data from the sample size used in this study was enough to demonstrate significant differences between groups.
Data exclusions	No datasets were excluded from analysis in this work.
Replication	All attempts at replication were successful. The cells used in these experiments come from at least 6 different human HSPC cell donors, and 4 different WAS patients. All the experiments were performed with at least 3 replicates. Mouse transplantation was performed with HSPCs derived from one WAS patient and 2 healthy donors, with two experimental replicates each. For the off-target analysis, two human cell donors were used.
Randomization	For wild type control, cells were derived exclusively from male healthy donors since WAS pathogenesis affects only male infants. The cells derived from WAS patients with different pathogenic mutation were random and was based on the availability of such samples in the hospital. Mice were randomly assigned to each experimental group and cage cohorts always consisted of mixed experimental conditions.
Blinding	Podosome counting in confocal analysis was performed in blinded fashion. Immunofluorescence slides and images were coded by a lab member who was not involved in this study. Mice were analyzed in a blinded fashion. Mice were tagged with a code after transplantation and all the investigators were blinded during harvesting of tissues and data recording. For all in vitro experiments, the lead investigator was aware of the treatment group as it was very important to know clear difference between samples for downstream assays. To remove any biases, the lead investigator had appropriate positive and negative control samples with standard protocol applied equally in all the samples.

Reporting for specific materials, systems and methods

We require information from authors about some types of materials, experimental systems and methods used in many studies. Here, indicate whether each material, system or method listed is relevant to your study. If you are not sure if a list item applies to your research, read the appropriate section before selecting a response.

Materials & experimental systems

n/a	Involved in the study	
☐	☒	Antibodies
☐	☒	Eukaryotic cell lines
☒	☐	Palaeontology
☐	☒	Animals and other organisms
☐	☒	Human research participants
☒	☐	Clinical data

Methods

n/a	Involved in the study	
☒	☐	ChIP-seq
☐	☒	Flow cytometry
☒	☐	MRI-based neuroimaging

Antibodies

Antibodies used	Mouse anti-human CD45 APC HI30 BD Bioscience 555485<br>Mouse anti-human CD45 BV 421 HI30 Biolegend 304032

Mouse anti-human CD3 PE OKT3 Biolegend 317308
Mouse anti-human CD33 FITC P67.6 BD Bioscience 345798
Mouse anti-human CD19 PerCp Cy5.5 HIB19 Biolegend 302230
Mouse anti-human WASP - 5A5 BD Bioscience 557773
Goat anti-mouse IgG Alexa Fluor 647 Poly4053 Biolegend 405322
Rabbit anti-human WASp - EP2541Y Abcam ab75830
Donkey anti-rabbit WASp Alexa Fluor 647 Poly4064 Biolegend 406414
Mouse anti-human CD34 BV 421 561 Biolegend 343610
Mouse anti-human CD34 FITC 561 Biolegend 343604
Mouse anti-human CD38 APC Cy7 HIT2 Biolegend 303534
Mouse anti-human CD90 PE Cy7 5E10 Biolegend 328124
Mouse anti-human CD45RA APC HI100 Biolegend 304112
Mouse anti-human CD45RA PerCp Cy5.5 HI100 Biolegend 304122
Mouse anti-human CD41a FITC HIP8 Biolegend 303704
Mouse anti-human CD42b APC HIP8 BD Bioscience 551061
Mouse anti-human CD61 APC VI-PL2 Biolegend 336412
Mouse anti-human CD62p PE VI-P44 Biolegend 304906
Mouse anti-human CD3 - OKT3 Biolegend 317302
Mouse anti-human Vinculin - V4505 Sigma-Aldrich V4505
Goat anti-mouse IgG Alexa Fluor 488 H+L ThermoFisher Scientific A28175
Annexin V APC BD Bioscience 550474
Mouse anti-human GAPDH 0411 Santa Cruz sc-47724
Sheep anti-mouse IgG HRP GE Healthcare NXA931
Mouse anti-human CD14 PerCp Cy5.5 G1D3 ThermoFisher Scientific 45-0149-42
Mouse anti-human CD16 APC CB16 ThermoFisher Scientific 17-0168-41
Phalloidin Alexa Fluor 635 ThermoFisher Scientific A34054

Validation

All antibodies used here have been previously reported and are routinely used in flow cytometry studies. All the antibodies used in this study were routinely tested in flow cytometry. Antibodies were validated by using positive (antigen positive cells) and negative (isotype and/or antigen negative cells) controls with the recommended antibody concentrations from the manufacturers. All the antibodies used here are already listed in LABOME website (https://www.labome.com/index.html), which contains database for all the validated antibodies from previous publications. A literature reference link is provided for each antibody used.

Mouse anti-human CD45 APC HI30 BD Bioscience 555485 (Ref: https://www.ncbi.nlm.nih.gov/pmc/articles/PMC6477023/)
Mouse anti-human CD45 BV 421 HI30 Biolegend 304032 (Ref: https://www.ncbi.nlm.nih.gov/pmc/articles/PMC6559950/)
Mouse anti-human CD3 PE OKT3 Biolegend 317308 (Ref: https://www.ncbi.nlm.nih.gov/pmc/articles/PMC6514199/)
Mouse anti-human CD33 FITC P67.6 BD Bioscience 345798 (Ref: https://ashpublications.org/blood/article-lookup/doi/10.1182/blood-2017-02-768762)
Mouse anti-human CD19 PerCp Cy5.5 HIB19 Biolegend 302230 (Ref: https://www.ncbi.nlm.nih.gov/pmc/articles/PMC6544433/)
Mouse anti-human WASP - 5A5 BD Bioscience 557773 (Ref: https://www.ncbi.nlm.nih.gov/pmc/articles/PMC6721834/)
Goat anti-mouse IgG Alexa Fluor 647 Poly4053 Biolegend 405322 (Ref: https://www.ncbi.nlm.nih.gov/pmc/articles/PMC6459881/)
Rabbit anti-human WASp - EP2541Y Abcam ab75830 (Ref: https://www.abcam.com/waspwiskott-aldrich-syndrome-protein-antibody-ep2541y-ab75830.html)
Donkey anti-rabbit WASp Alexa Fluor 647 Poly4064 Biolegend 406414 (Ref: https://www.ncbi.nlm.nih.gov/pmc/articles/PMC6454047/)
Mouse anti-human CD34 BV 421 561 Biolegend 343610 (Ref: https://www.ncbi.nlm.nih.gov/pmc/articles/PMC6436882/)
Mouse anti-human CD34 FITC 561 Biolegend 343604 (Ref: https://www.ncbi.nlm.nih.gov/pmc/articles/PMC6323118/)
Mouse anti-human CD38 APC Cy7 HIT2 Biolegend 303534 (Ref: https://www.ncbi.nlm.nih.gov/pmc/articles/PMC3273988/)
Mouse anti-human CD90 PE Cy7 5E10 Biolegend 328124 (Ref: https://www.cell.com/cell-reports/fulltext/S2211-1247(19)30864-2?_returnURL=https%3A%2F%2Flinkinghub.elsevier.com%2Fretrieve%2Fpii%2FS2211124719308642%3Fshowall%3Dtrue)
Mouse anti-human CD45RA APC HI100 Biolegend 304112 (Ref: https://www.ncbi.nlm.nih.gov/pmc/articles/PMC5490755/)
Mouse anti-human CD45RA PerCp Cy5.5 HI100 Biolegend 304122 (Ref: https://www.ncbi.nlm.nih.gov/pmc/articles/PMC6350210/)
Mouse anti-human CD41a FITC HIP8 Biolegend 303704 (Ref: https://www.ncbi.nlm.nih.gov/pmc/articles/PMC6007942/)
Mouse anti-human CD42b APC HIP8 BD Bioscience 551061 (Ref: https://www.ncbi.nlm.nih.gov/pmc/articles/PMC4674320/)
Mouse anti-human CD61 APC VI-PL2 Biolegend 336412 (Ref: https://www.ncbi.nlm.nih.gov/pmc/articles/PMC6185941/)
Mouse anti-human CD62p PE VI-P44 Biolegend 304906 (Ref: https://www.ncbi.nlm.nih.gov/pmc/articles/PMC5052631/)
Mouse anti-human CD3 - OKT3 Biolegend 317302 (Ref: https://www.nature.com/articles/s41586-019-0964-2)
Mouse anti-human Vinculin - V4505 Sigma-Aldrich V4505 (Ref: https://www.ncbi.nlm.nih.gov/pmc/articles/PMC5403263/)
Goat anti-mouse IgG Alexa Fluor 488 H+L ThermoFisher Scientific A28175 (Ref: https://www.ncbi.nlm.nih.gov/pmc/articles/PMC7018987/)
Annexin V APC BD Bioscience 550474 (Ref: https://www.ncbi.nlm.nih.gov/pmc/articles/PMC6078823/)
Mouse anti-human GAPDH 0411 Santa Cruz sc-47724 (Ref: https://www.ncbi.nlm.nih.gov/pmc/articles/PMC6782013/)
Sheep anti-mouse IgG HRP GE Healthcare NXA931 (Ref: https://www.ncbi.nlm.nih.gov/pmc/articles/PMC6909970/)
Mouse anti-human CD14 PerCp Cy5.5 G1D3 ThermoFisher Scientific 45-0149-42 (Ref: https://www.ncbi.nlm.nih.gov/pmc/

articles/PMC5760367/)
Mouse anti-human CD16 APC CB16 ThermoFisher Scientific 17-0168-41 (Ref: https://www.ncbi.nlm.nih.gov/pmc/articles/PMC5783502/)
Phalloidin Alexa Fluor 635 ThermoFisher Scientific A34054 (Ref: https://www.ncbi.nlm.nih.gov/pmc/articles/PMC5471308/)

Eukaryotic cell lines

Policy information about cell lines

Cell line source(s)	HEK293T
Authentication	HEK293T cell line was obtained from ATCC (https://www.lgcstandards-atcc.org/products/all/crl-3216.aspx?geo_country=gb#documentation). ATCC used morphology, PCR and karyotyping to authenticate HEK293T cell line.
Mycoplasma contamination	The cell line tested negative for mycoplasma contamination
Commonly misidentified lines (See ICLAC register)	No commonly misidentified cell lines were used in this study

Animals and other organisms

Policy information about studies involving animals; ARRIVE guidelines recommended for reporting animal research

Laboratory animals	Species: Mus musculus<br>Strains:<br>1) NOD.Cg-PrkdcscidIL2rgtmlWjl/Sz (NSG) mice were developed at The Jackson Laboratory.<br>Age:<br>For all studies mice were transplanted at 6-8 weeks. Primary engraftement was measured 14 weeks post-transplantation, secondary transplants were analyzed after an additional 12 weeks (total 26 weeks). Biochemical and phenotypic correction was measured between 14-26 weeks post-transplant.<br>Transplants were performed in females mice.
Wild animals	Wild animals were not used.
Field-collected samples	The study did not involve samples collected from the field.
Ethics oversight	For experiments involving animals, mice were bred and maintained in accordance with UK Home Office regulations, and experiments were conducted after approval by the University College London Animal Welfare and Ethical Review Body (project license 70/8241).

Note that full information on the approval of the study protocol must also be provided in the manuscript.

Human research participants

Policy information about studies involving human research participants

Population characteristics	HSPCs and T cells were isolated from de-identified healthy human male donors
Recruitment	Before undergoing stem cell transplantation treatment, WAS patients agreed and signed informed consent to take their apheresis blood for research studies. No self-selection bias is anticipated.
Ethics oversight	Great Ormond Street Hospital for Children NHS Foundation and the Institute of Child Health Research Ethics (08/H0713/87)

Note that full information on the approval of the study protocol must also be provided in the manuscript.

Flow Cytometry

Plots

Confirm that:

- [x] The axis labels state the marker and fluorochrome used (e.g. CD4-FITC).
- [x] The axis scales are clearly visible. Include numbers along axes only for bottom left plot of group (a 'group' is an analysis of identical markers).
- [x] All plots are contour plots with outliers or pseudocolor plots.
- [x] A numerical value for number of cells or percentage (with statistics) is provided.

Methodology

Sample preparation	All detailed in material and methods. Different protocols for each antibody were used.

Instrument	BD FACSAria II (BD Bioscience) instrument was used for cell sorting of CD34+ HSPCs. For all flow cytometry analysis, a BD LSRII instrument (BD Bioscience) was used.
Software	BD FACSDIVA (Becton Dickinson) software was included in the BD FACSaria II and BD LSR II analyzer and was used to collect flow cytometry data. FlowJo v10.5 (FlowJo, LLC) software was used to analyse the data.
Cell population abundance	As expected, the abundance of HSCs (CD34+ CD38- CD90+ CD45RA-) and MPPs (CD34+ CD38- CD90- CD45RA-) were slightly lower than CD34+ CD38+ cell population within the post-sort fraction. However the purity of each sorted fraction were >99%. This was determined by taking a small volume of sample from the sorted cells and running again through BD FACSAria II instrument.
Gating strategy	The total cell population was gated sing FSC vs SSC plot. From this initial population, exclusion of doublet cells and dead cells were performed. After this, the positively stained cells (i.e. cells of interest) were gated using the negative control samples.

☒ Tick this box to confirm that a figure exemplifying the gating strategy is provided in the Supplementary Information.
